# Short- and long-term consequences of heat exposure on mitochondrial metabolism in zebra finches (*Taeniopygia castanotis*)

**DOI:** 10.1007/s00442-023-05344-7

**Published:** 2023-03-10

**Authors:** Hector Pacheco-Fuentes, Riccardo Ton, Simon C. Griffith

**Affiliations:** School of Natural Sciences, Macquarie University, Sydney, NSW 2109 Australia

**Keywords:** Acclimation, Carry-over effects, Developmental programming, Heatwave, Metabolic rates

## Abstract

Understanding the consequences of heat exposure on mitochondrial function is crucial as mitochondria lie at the core of metabolic processes, also affecting population dynamics. In adults, mitochondrial metabolism varies with temperature but can also depend on thermal conditions experienced during development. We exposed zebra finches to two alternative heat treatments during early development: “constant”, maintained birds at ambient 35 °C from parental pair formation to fledglings’ independence, while “periodic” heated broods at 40 °C, 6 h daily at nestling stage. Two years later, we acclimated birds from both experiments at 25 °C for 21 days, before exposing them to artificial heat (40 °C, 5 h daily for 10 days). After both conditions, we measured red blood cells’ mitochondrial metabolism using a high-resolution respirometer. We found significantly decreased mitochondrial metabolism for Routine, Oxidative Phosphorylation (OxPhos) and Electron Transport System maximum capacity (ETS) after the heat treatments. In addition, the birds exposed to “constant” heat in early life showed lower oxygen consumption at the Proton Leak (Leak) stage after the heat treatment as adults. Females showed higher mitochondrial respiration for Routine, ETS and Leak independent of the treatments, while this pattern was reversed for OxPhos coupling efficiency (OxCE). Our results show that short-term acclimation involved reduced mitochondrial respiration, and that the reaction of adult birds to heat depends on the intensity, pattern and duration of temperature conditions experienced at early-life stages. Our study provides insight into the complexity underlying variation in mitochondrial metabolism and raises questions on the adaptive value of long-lasting physiological adjustments triggered by the early-life thermal environment.

**Supplementary Information:**

The online version contains supplementary material available at 10.1007/s00442-023-05344-7.

## Introduction

High-ambient temperatures can generate stressful physiological conditions in most organisms (Eyck et al. 2019) including avian species adapted to inhabit arid and desert ecosystems (McKechnie and Wolf 2010). These thermal environments challenge the ability of individuals of all ages to maintain homeostasis and prevent heat stress that may have downstream detrimental consequences on population dynamics (Iknayan and Beissinger 2018) by increasing reproductive failure, reducing parental care, and lowering chicks growing rates, among others (Andreasson et al. 2018, 2020). In addition, it has been shown that the distribution of species may be dependent on the magnitude and predictability of climatic change interacting with evolved levels of plasticity in heat tolerance (Reed et al. 2010; Pollock et al. 2021). The physiological stability required to operate in different thermal regimes includes the modulation of mitochondrial activity, the subcellular organelles that lay at the core of every metabolic process and are responsible for energy turnover and heat production (Wisnovsky et al. 2016).

In the light of the current climate crisis and the associated higher occurrence of extreme temperatures (IPCC 2014), it is important to investigate how mitochondrial functions vary based on thermal conditions experienced during the lifetime of an organism. Despite the pivotal role that mitochondria play in determining individual fitness (Criscuolo et al. 2005), our understanding of the long- and short-term consequences that exposure to high temperatures may have on variation in mitochondrial metabolism is still limited (Nord and Giroud 2020). Recent observational and experimental studies have shown that exposure to environmental stresses during early-life stages can yield detrimental consequences on mitochondrial performance in adulthood (Eyck et al. 2019; Reynolds et al. 2019; Casagrande et al. 2020; Gyllenhammer et al. 2020). Environmental challenges during growth and development can also program organisms to better withstand those conditions in the future (Andreasson et al. 2018; Stier et al. 2022). Either detrimental, or adaptive physiological modifications due to environmental conditions during early development represent permanent changes that persist over time and can also potentially be inherited by future generations (Grilo et al. 2021). Many of the studies cited above have focussed on general stressors, and a key question in the field of thermal ecology is the extent to which variation in mitochondrial metabolism may arise from the interplay between temperatures experienced during growth and development, and those that will occur in adulthood.

To date, most experimental avian research on the response of mitochondria to heat has focussed on domestic species of poultry (Mujahid et al. 2006, 2007; Tan et al. 2010; Zhang et al. 2016). These studies provide a useful and extensive background to mitochondrial physiology (Huang et al. 2015; Akbarian et al. 2016), but domestic poultry experience strong artificial selection and live in tightly controlled temperature regimes (Wasti et al. 2020), therefore, permitting limited ecological inference of relevance to wild species. In addition, most evidence of long-term modifications induced by heat on mitochondrial metabolism comes from studies that have used destructive sampling and thus were unable to compare the same individuals over time (Loyau et al. 2014, 2015). A more stringent test may come from repeated measurements of the same subjects in different thermal conditions, but these have been scarce to date due to intrinsic limitations associated with the often-destructive sampling methods in studies of mitochondrial function (Stier et al. 2022). Birds provide a good opportunity to overcome those limitations because their erythrocytes have functionally active mitochondria (Stier et al. 2013). This allows for minimally invasive repeated sampling over time and circumvents the need for destructive protocols or complex muscle and organ biopsies that can compromise health and survival (Stier et al. 2015, 2017).

One of the few available studies examining the consequences of heat stress on wildlife recorded higher Baseline, Oxidative Phosphorylation (OxPhos) and Electron Transport System maximum capacity (ETS) 20 days post-treatment for eggs that were incubated at high temperatures (Stier et al. 2022). A second study reported that heat stress effects during the post-natal stage increased mitochondrial metabolism, for the Baseline and Proton Leak (Leak) stages of mitochondrial respiration 70 days after the treatment was ended (Ton et al. 2021). These recent findings (Ton et al. 2021; Stier et al. 2022), suggest that heat during growth and development can influence mitochondrial metabolism in the short term, but the existence of mitochondrial modifications over longer time frames is yet to be documented. This gap in knowledge is important to address because the programming hypothesis is grounded on the prediction that environmental influences on the phenotype of developing embryos should lead to life-long modifications, that may trade-off with long-term costs (Gyllenhammer et al. 2020). A second prediction related to the potential adaptive value of the programming hypothesis is that adjustments in mitochondrial functions in adulthood should be triggered by those same stressful conditions experienced during early life and embryonic development (Gyllenhammer et al. 2020). Indeed, acute stressors are expected to favour the emergence of reversible phenotypic plasticity that, over the lifetime of an organism, should be triggered by reliable cues as an adaptive strategy to cope with environmental variability (Gabriel 2005).

To investigate the predictions outlined above, we used zebra finches, a native Australian species adapted to arid environments, which is also one of the most commonly used model species for laboratory tests all over the world (Griffith et al. 2021). Australia is predicted to witness some of the most extreme heatwaves on the planet in coming decades (Cowan et al. 2014; Jyoteeshkumar reddy et al. 2021), and, therefore, the zebra finch provides an ideal species to assess the physiological boundaries of mitochondrial functions experimentally. We exposed two groups of birds to one of two alternative experiments early in their life. One group was kept in a temperature-controlled room, receiving constant heat from the time that their parents formed pairs, through the reproductive cycle and until the focal individuals reached independence (hereafter referred to as ‘constant heat’). The other experimental group was only exposed to heat intermittently during their nestling stage (hereafter ‘periodic heat’). Both of these experimental groups also had a control group that were produced concurrently under control thermal conditions. To investigate whether consequences of early thermal conditions carry over to adulthood, 2 years later, mitochondrial respiration of red blood cells was measured on the experimental birds that were produced under these manipulations (and their respective controls) both before and after experiencing an experimental heatwave as adults. This design allowed us to test acclimation processes in mitochondrial metabolism at different temperatures in adulthood and examine the possibility that variation in mitochondrial functions in adulthood can be causally linked to differences between our early-life experiments in the duration, intensity, and pattern of temperature exposure.

## Materials and methods

### Experiments in early-life stages

All birds used in the present study were produced from a captive population of zebra finches (*Taeniopygia castanotis*) originally taken from the wild between 3 and 5 generations prior to this work at Macquarie University (Sydney) in 2018.

#### Periodic heat experiment

**Fig. 1:**
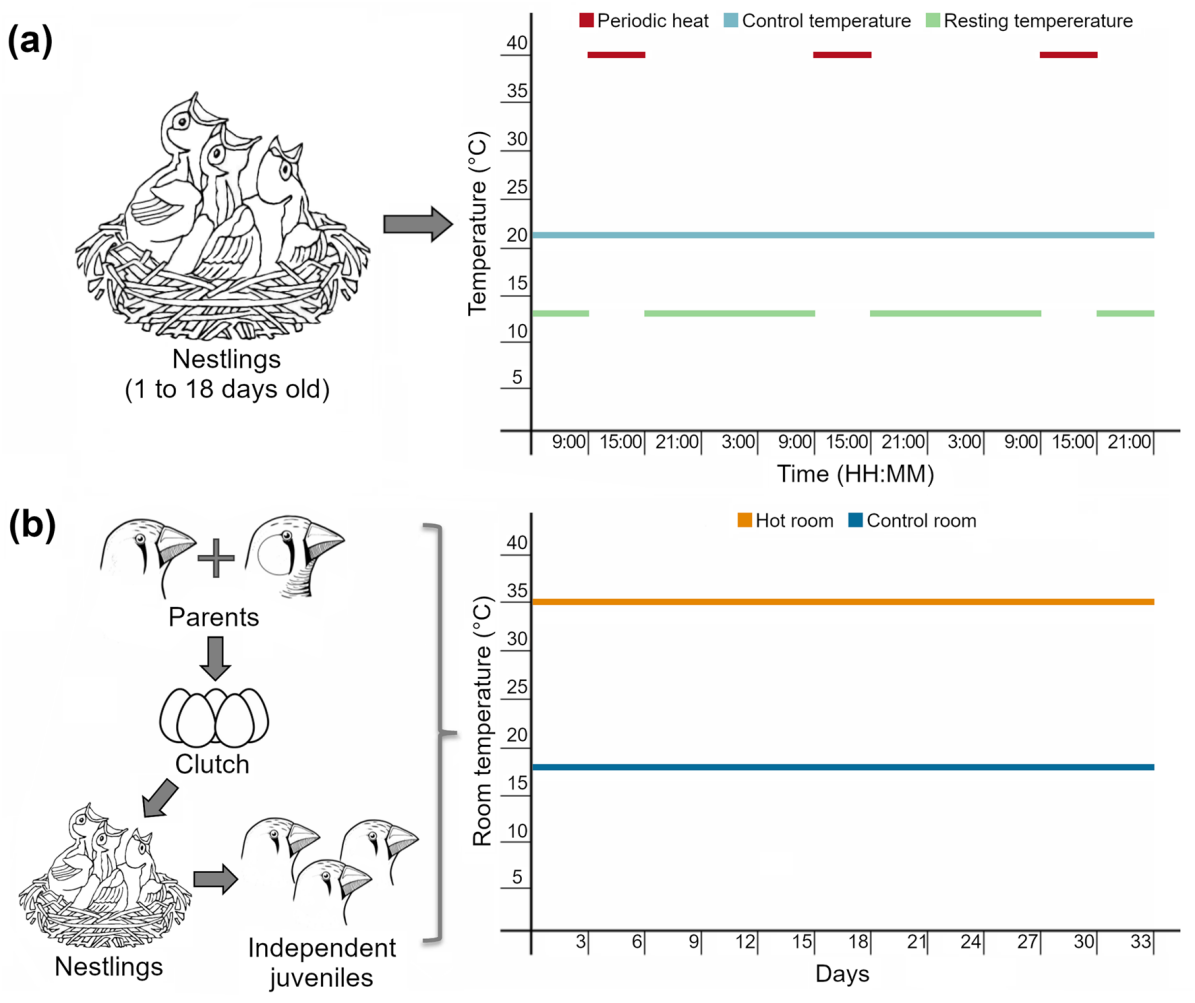
Early-life treatments scheme, showing the (**a**) periodic heat experiment, where zebra finches broods were heated during the nestling stage with conductive hot plates (40 ± 0.2 °C) for 6 h day (i.e. periodic heat, red dashed line), and experienced a mean ambient temperature of 13.21 ± 0.38 °C during the 18 h of the day they were not receiving the treatment (i.e. resting temperature, green dashed line). A group of nestlings from other broods were maintained at ambient temperature (21.5 ± 1.84 °C) as controls (i.e. turquoise line); and (**b**) constant heat experiment, where the birds were kept at the constant temperature of either 18.5 ± 0.04 °C (i.e. control room, blue line), or 34.7 ± 0.01 °C (i.e. hot room, orange line) from parental pair formation to independence. Coloured lines indicate mean temperature ± SE

The periodic heat experiment was conducted during the nestling stage only (i.e. 1 day post-hatch until day 18 of the nestling stage). The nests of 14 individual nestlings from 11 independent broods were heated with conductive hot plates (40 ± 0.2 °C) for 6 h a day between 9:00 and 15:00 (i.e. periodic heat), and experienced a mean ambient temperature of 13.21 ± 0.38 °C during the 18 h they were not receiving the heat treatment (i.e. resting temperature). Nineteen nestlings from 11 other broods were kept at ambient temperature (21.5 ± 1.84 °C) as controls (i.e. control temperature) (Fig. 1a; Supplementary Material, Table 1; see Ton et al. 2021 for further methodological details).

#### Constant heat experiment

In the constant heat experiment, the thermal conditions experienced were manipulated for the whole breeding cycle from the pairing of adults, through to the independence of the focal individuals as offspring (i.e. at 36 days after hatching). Twenty pairs of adult zebra finches were allowed to breed in controlled temperature rooms at the constant temperature of either 18.5 ± 0.04 °C (control, from now on 18 °C), or 34.7 ± 0.01 °C (heat treatment, from now on 35 °C) (Fig. 1b; Supplementary Material, Table 1).

### Treatments in adulthood

The results reported in the present study were generated using 65 birds (37 males and 28 females) from both early-life experiments that we maintained in our study population to adulthood (see Supplementary Material, Table 2). Twenty-six of these 65 individuals (13 males and 13 females) were produced in the periodic heat experiment (14 heated and 12 controls), while the 39 remaining birds (24 males and 15 females) came from the constant heat experiment (19 heated and 20 controls). Adult birds ranged between 19 and 26 months of age, mean ± SE age was 23.8 ± 0.18 and 22.21 ± 0.34 months for the periodic and constant heat experiments, respectively. All the birds were in apparent good health and not showing any detrimental consequences of their early-life heat exposure at the time of their treatment in adulthood and we did not experience any casualty following our treatments. These adult zebra finches were randomly grouped in ten sets of six and one set of five same sex individuals in large cages (70 × 47 × 30 cm in height, width, and depth, respectively), and held inside Controlled Temperature Rooms (CTRs) at 24.8 ± 0.01 °C (from now on 25 °C) for 3 weeks, during which they were provided with dry finch seed mix (AviGRAIN, Finch Blue mix), and water ad libitum, supplemented with cuttlebone and shell grit in 12 h 30 min daylight, 11 h 30 min of darkness cycles. Each cage contained a random mix of individuals from the two different treatments and the controls. At the end of this period, all birds were exposed to a heat treatment where they experienced a mean ± SE temperature of 40 ± 0.02 °C (hereafter 40 °C), 5 h daily from 10:00 to 15:00 for ten consecutive days. This temperature was chosen to emulate heatwave-like conditions that have been recorded in the natural habitat of the zebra finch (Griffith et al. 2016, 2020; Cooper et al. 2020a). In addition, this temperature is unlikely to be too harmful for the birds as demonstrated in previous in-lab studies (Cooper et al. 2020b; Ton et al. 2021; Udino et al. 2021), but it challenges the birds to maintain physiological homeostasis as it is above the recently re-defined thermoneutral zone for the species (Wojciechowski et al. 2021). To avoid temperature gradients, generic 40 cm pedestal fans were installed inside CTRs to constantly redistribute the hot air evenly across the room (the temperature across the room was checked using iButtons). For the remaining 19 h of the days on which birds were not exposed to the heat treatment, the birds were moved into another CTR set at 24.8 ± 0.11 °C. Every bird was provided with dry finch seed mix (AviGRAIN, Finch Blue mix) while exposed to heat, but were water deprived during the treatment, as otherwise an increased ingestion of water would reduce the physiological efficacy of the treatment (Funghi et al. 2019; Cooper et al. 2020b; Pacheco-Fuentes et al. 2022). Water was immediately provided after the daily period of elevated heat, to avoid stress-related heat shock (Cooper et al. 2020b).

### Mitochondrial respiration measurements

Mitochondrial respiration was measured at 37 °C on every bird (following Stier et al. 2013), after the acclimation period at 25 °C, and then again 1 day after the end of the 10-day heat treatment (40 °C). We picked the assay temperature based on previous studies of the zebra finch (Stier et al. 2013), which also allowed us to compare our dataset with previous datasets obtained from the same experimental subjects (Ton et al. 2021). Given the time taken to conduct each assay, groups of three randomly chosen birds were moved into the heated room each day. By staggering the beginning of the heat treatment, after ten consecutive days of heat, three birds were ready to be sampled each day. For the mitochondrial assay, 60–70 µl blood samples were collected from each birds’ brachial vein in duplicates, using heparinised capillaries. The blood samples were then transferred to 1.5 ml Eppendorf tubes and centrifuged for 5 min at 3,000 rpm in a Sigma 1–14 centrifuge, to separate red blood cells (RBCs) from plasma (following Stier et al. 2017). After centrifugation, the RBCs pellets were gently mixed three times with 1 ml of ice-cold phosphate buffered saline (PBS 0.4%, pH 7.4). Then, the mixes were centrifuged at 800 rpm for 3 min to eliminate plasma remnants, discarding the supernatant, and keeping the RBCs pellets.

Prior to measuring mitochondrial respiration, each RBCs pellet was gently resuspended in 1 ml of MiR05 medium (0.5 mM Egtazic Acid (EGTA), 3 mM MgCl2, 60 mM K-lactobionate, 20 mM taurine, 10 mM KH2PO4, 20 mM Hepes, 110 mM sucrose, free fatty acid bovine serum albumin (1 g L^−1^), pH 7.1; Oroboros Instruments, Innsbruck, Austria) taken from one of the two available high-resolution respirometer chambers (O2k, Oroboros Instruments, Innsbruck, Austria). The resuspended pellet (1 ml) was then transferred back into each chamber. Once the O2k sensor stabilised (~ 10 min), the following parameters were measured: a) basal mitochondrial oxygen consumption (Routine). Mitochondrial respiration was then boosted by adding 5 µl of 2 mM pyruvate; b) mitochondrial oxygen consumption related to Proton Leak (Leak) after stepped-titrating 2.5 µl of 0.5 mM oligomycin (i.e. ATP production inhibitor; 1 µl first, then three 0.5 µl injections every 5 min until reaching signal’s stability); c) Oxidative Phosphorylation (OxPhos) was obtained as result of subtracting Leak from Routine; d) Electron Transport System (ETS) maximum capacity using the uncoupler carbonyl cyanide m-chlorophenyl hydrazine (CCCP) 1 mM, 1 µl titrations until reaching maximum uncoupled state (~ 6 µl); and e) mitochondrial respiration inhibition (i.e. complex III inhibition) using 5 µl of 1 mM antimycin A, to quantify and further subtract non-mitochondrial respiration from the previous measured steps (Stier et al. 2017). Final concentrations of pyruvate, oligomycin, CCCP and antimycin A were 5, 0.625, 3 and 2.5 µmol L^−1^, respectively. After each run, 1 ml per chamber (i.e. MiR05 + RBCs) was collected and kept at − 80 °C to further measure protein concentration through bicinchoninic acid assay (Pierce BCA protein quantification assay; ThermoFisher Scientific, Waltham, MA, United States), considered an accurate method to estimate cellular density in birds’ blood samples, and is highly correlated with the number of cells being measured (Stier personal communication, 28-Jul-2022). Mitochondrial respiration stages were then normalised by including the sample protein content as a covariate in the statistical analyses. Two mitochondrial flux ratios were calculated to test mitochondrial efficiency pre- and post-heat treatment: OxPhos coupling efficiency [OxCE = 1 − (Leak/Routine)], and mitochondrial reserve capacity (FCR_R/ETS_ = Routine/ETS) (Ton et al. 2021). The OxCE calculates the fraction of the routine respiration that can be linked to ATP synthesis, as such it can be used as a proxy of mitochondrial ATP production. The second ratio (FCR_R/ETS_) gives the fraction of ETS maximum respiratory capacity used by the cell under routine conditions. It is a measure of the oxidative reserve capacity, which tells us how far below its maximum the cell, is operating. An individual with a high reserve capacity has more flexibility to respond to extra energy demands. In addition, the technical replicates obtained from the trials (i.e. two respirometer chambers per each bird blood sample) were used to calculate the intra-class coefficients of correlation (ICC; 0.55–0.78, *p* < 0.001 for all stages measured) in order to determine the repeatability of the mitochondrial respiration stages measured (Ton et al. 2021).

### Statistical analysis

The statistical analyses were conducted in R version 4.1.2 (R Core Team 2021) for Microsoft Windows, employing RStudio version 1.2.5033 (RStudio Team 2020) as the graphical user interface.

Preliminary analyses were performed to determine the normal distribution of the data, normality of residuals, influential observations, homogeneity of variance, and to detect collinearity between variables of interest (see Supplementary Material). To evaluate the presence/absence of interaction effects between the treatment in adulthood and the early-life experiments on mitochondrial metabolism, we followed a model comparison approach using AIC (Aho et al. 2014; see Supplementary Material, Section 4). We compared a set of five different linear mixed models (LMMs) (lme4 and lmerTest R packages; Bates et al. 2014; Kuznetsova et al. 2017) formalised according to the research hypothesis (see Supplementary Material, Section 4). Each one of the mitochondrial respiration stages measured was used as dependent variables and evaluated separately. Sex and sample mass in the form of protein content were included in all models, but protein content was excluded when testing OxCE and FCR_R/ETS_ because these values are ratios. Bird IDs were included as a random factor to account for individual variability and repeated measures.

## Results

### Model selection

Our model comparison approach identified model four to have the lowest AIC and, therefore, to be the model of choice to best describe the available data for Routine (AIC_weight_ = 51%), Leak (AIC_weight_ = 85%) and the mitochondrial flux control ratios (FCR _R/ETS_ (AIC_weight_ = 73%); OxCE (AIC_weight_ = 65%)). This provides evidence for the role of an interaction between the thermal conditions experienced in early life and treatments in adulthood in determining mitochondrial respiration (see Appendix A, Tables 1, 3, 9 and 11). Conversely, model two was preferred for ETS (AIC_weight_ = 39%) and OxPhos (AIC_weight_ = 78%) suggesting that only the treatment in adulthood had a significant effect on the dependent variables (see Appendix A, Tables 5 and 7).

### Effects of protein content and sex

Protein content was a significant covariate in all our models, and showed a positive correlation with the four mitochondrial respiration stages measured in adulthood (Routine: *F*_(216.56)_ = 54.15, *P* < 0.001; Leak: *F*_(214.17)_ = 39.14, *P* < 0.001; OxPhos: *F*_(226.54)_ = 39.40, *P* < 0.001; and ETS: *F*_(225.74)_ = 7.97, *P* = 0.005; see Appendix A, Tables 2, 4, 6 and 8), as expected under metabolic theory (West et al. 2002). Females showed consistently higher rates of oxygen consumption compared to males for Routine (*F*_(60.85)_ = 7.26, *P* = 0.009), Leak (*F*_(60.82)_ = 14.28, *P* < 0.001), and ETS (*F*_(63.73)_ = 5.01, *P* = 0.03). This pattern was reversed for OxCE (*F*_(59.84)_ = 5.43, *P* = 0.02). We detected no differences between the sexes for OxPhos (*F*_(63.74)_ = 1.78, *P* = 0.19) and FCR _R/ETS_ (*t*_(59.30)_ = 0.34, *P* = 0.562).

### Short-term temperature effects on mitochondrial respiration stages in adult birds

**Fig. 2:**
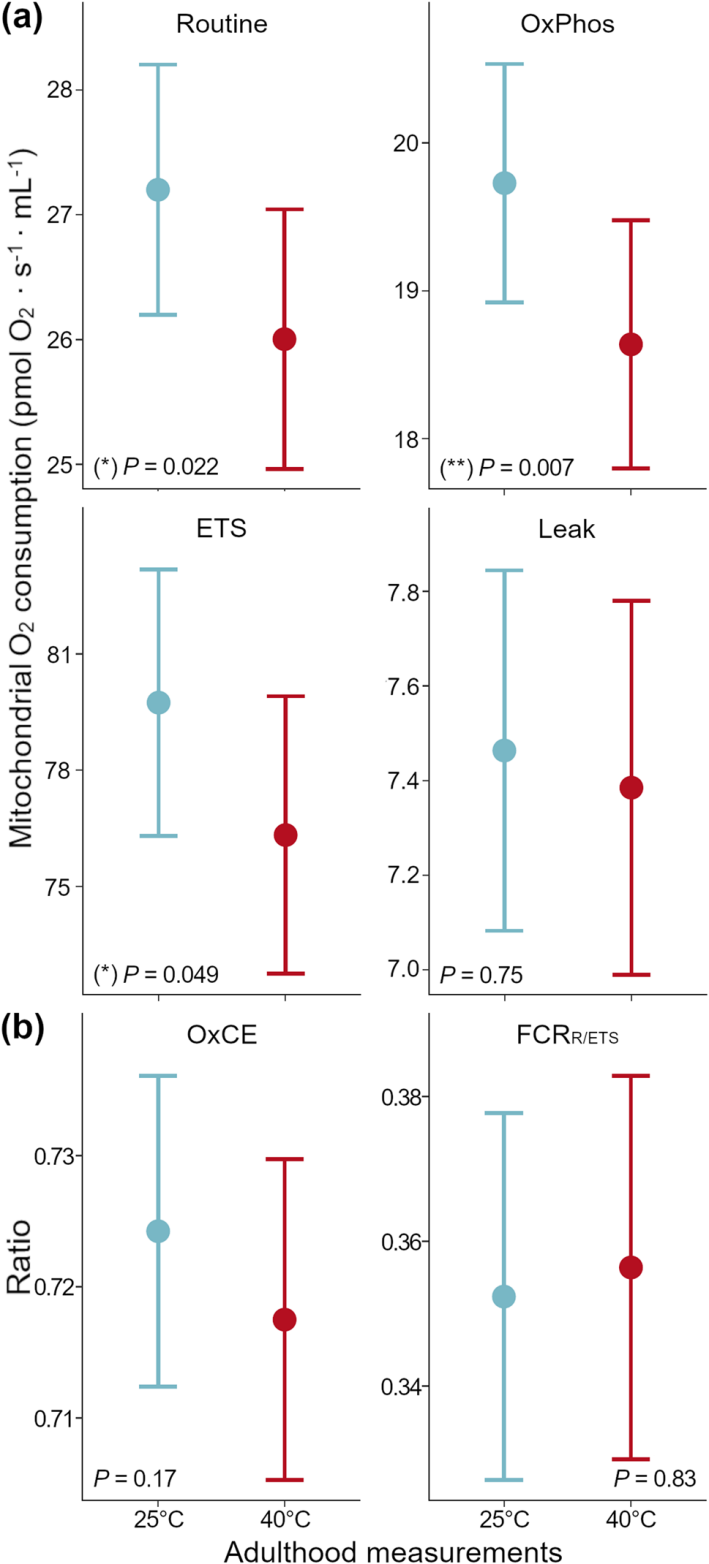
Effects on (**a**) mitochondrial respiration stages of adult zebra finches, measured post-acclimation at 25 °C and after 10 days of heat treatment at 40 °C (i.e. adulthood measurements), where Routine represents the basal mitochondrial physiological activity; OxPhos the mitochondrial respiration stage associated with ATP production; ETS the mitochondrial maximum respiratory capacity; Leak the mitochondrial respiration backwards proton flow from membrane to mitochondrial matrix; and (**b**) mitochondrial flux ratios of adult zebra finches, also measured post-acclimation at 25 °C, and after 10 days of exposure at 40 °C (i.e. adulthood measurements)**,** where OxCE is OxPhos coupling efficiency; and FCR_R/ETS_ represents the mitochondrial reserve capacity. Dots and bars indicate mass-independent mean oxygen consumption ± SE

After being exposed to 40 °C for 5 h a day during ten consecutive days, adult birds showed a significant decrease in Routine (*F*_(180.47)_ = 5.30, *P* = 0.022), OxPhos (*F*_(183.59)_ = 7.42, *P* = 0.007) and ETS maximum capacity (*F*_(183.79)_ = 3.93, *P* = 0.049) compared to measurements performed after the 25 °C acclimation period (Fig. 2a; Appendix A, Tables 2, 6, and 8). Leak stage oxygen consumption did not show significant changes as a result of the heat challenge (*F*_(180.89)_ = 0.10, *P* = 0.75; Fig. 2a), similarly to what we recorded for the mitochondrial flux ratios OxCE and FCR _R/ETS_ (*F*_(173.14)_ = 1.93, *P* = 0.17, and *F*_(176.66)_ = 0.05, *P* = 0.83, respectively; Fig. 2b; Appendix A, Tables 4, 10 and 12).

### Interaction between heat exposure in early life and in adulthood

**Fig. 3:**
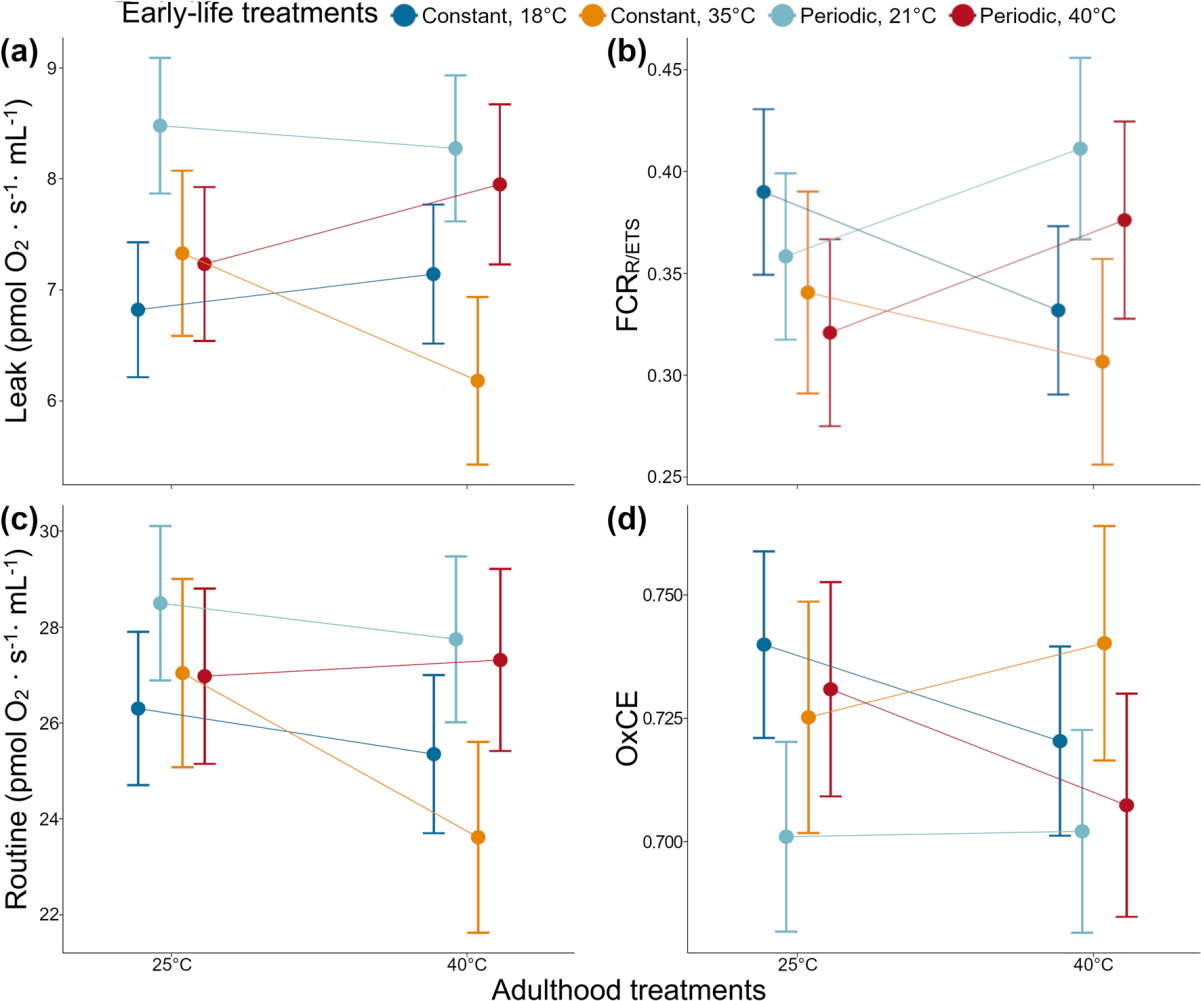
Interaction between early-life temperature conditions (i.e. early-life treatments) and exposure to different temperatures (25 and 40 °C) in adulthood (i.e. adulthood measurements) where (**a**) Leak (the backwards proton flow across the membrane into the mitochondrial matrix during respiration), and (**b**) FCR_R/ETS_ (the amount of extra ATP produced by oxidative phosphorylation to match an increase in energy demand), showed a significant interaction between early-life and adulthood treatments (*F*_(173.68)_ = 4.03, *P* = 0.008, and *F*_(175.82)_ = 4.22, *P* = 0.007, respectively). Both (**c**) Routine (basal mitochondrial oxygen consumption) and (**d**) OxCE (oxidative phosphorylation (OxPhos) coupling efficiency) showed no significant interaction between early-life and adulthood treatments but were slightly above the significance threshold (*F*_(173.40)_ = 2.31, *P* = 0.08; and *F*_(172.57)_ = 2.45, *P* = 0.07, respectively). Dots and bars indicate mass-independent mean oxygen consumption ± SE for each early-life treatment, while lines show the magnitude and direction of change in mitochondrial respiration between the different treatments that birds received during their adulthood

We detected a significant interaction between heat treatments at different life stages for Leak (*F*_(173.68)_ = 4.03, *P* = 0.008, Fig. 3a; Appendix A, Table 4). In particular, during this stage of mitochondrial respiration, individuals that were reared at constant heat (i.e. 35 °C), when re-exposed to 40 °C in their adulthood, showed a reduced oxygen consumption compared to birds that were periodically heated at 40 °C (*t*_(109.52)_ = -3.35, *P* = 0.019) or exposed to 21 °C in early life (*t*_(113.15)_ = 4.12, *P* = 0.001), but did not differ from their own control (*t*_(112.92)_ = 1.94, *P* = 0.48). In addition, the periodically heated birds when exposed to 40 °C in adulthood did not differ from their respective control group (*t*_(118.69)_ = 0.66, *P* = 0.99), and they did not differ from the constant control group either (*t*_(116.19)_ = -1.69, *P* = 0.65).

In addition, FCR _R/ETS_ showed a significant interaction between early life and adult treatments (*F*_(175.82)_ = 4.22, *P* = 0.007; Fig. 3b; Appendix A, Table 12). The post hoc tests indicated that when exposed to 40 °C in adulthood the birds that experienced the constant temperature of 35 °C in early life showed significantly lower values compared to those from the periodic experiment that were exposed to 21 °C (*t*_(141.80)_ = 3.06, *P* = 0.04).

Interestingly, while our model comparison process suggested the presence of a significant interaction between treatments at different life stages for Routine and OxCe (see “Results”, “Model selection”, and Appendix A, Tables 1 and 9), our analysis of deviance indicated otherwise with values slightly above the threshold for significance (Routine, *F*_(173.40)_ = 2.31, *P* = 0.08; OxCE, *F*_(172.57)_ = 2.45, *P* = 0.07; Fig. 3c, d, respectively; see Appendix A, Tables 2 and 10).

## Discussion

The present study represents one of the few available investigations to date about both short- and long-term consequences of heat exposure for different stages of mitochondrial metabolism in birds. Similar investigations are still in their infancy and are needed to fill a gap in our understanding of environmentally driven variation in mitochondrial responses (Chung and Schulte 2020; Nord et al. 2021). The results demonstrate that when adults were subjected to an experimental heatwave, the short-term response was to reduce oxygen consumption during Routine, OxPhos and ETS. This is consistent with some previous results (Tan et al. 2010; Guderley and Seebacher 2011) and confirms the plasticity of mitochondrial function (Stier et al. 2019). If blood mitochondrial metabolism mirrors whole body oxygen consumption, our results will be aligned with the avian literature on whole body metabolism, which has shown lower oxygen consumption when birds are acclimated to higher temperatures (Klaassen et al. 2004; McKechnie et al. 2007; Zheng et al. 2008). However, our study only considers red blood cells and, even though a mitochondrial metabolic correlation has been demonstrated with other tissues (Larsen et al. 2011; Stier et al. 2017), we acknowledge that the thermo-modulation role of blood remains poorly understood, particularly under stressful conditions (Malkoc et al. 2021).

Independently from the effects of treatments, we also detected a role of gender in driving variation in mitochondrial respiration with females showing higher oxygen consumption compared to males in three out of the four stages of mitochondrial respiration measured. These results replicate previous findings in other vertebrate (Viña et al. 2003), and invertebrate (Ballard et al. 2007) model species. Conversely, males showed higher OxCE. These results might contribute to gender differences in longevity caused by higher production of reactive oxygen species due to increased cellular respiration as suggested by the free oxygen radical theory of ageing (Harman 1956). However, that would require further exploration and the current study does not provide the data to examine this.

Our main finding was the presence of carry-over effects, of heat exposure early in development, on the mitochondrial metabolic response to heat about 2 years after one of our experiments. This result aligns with available evidence from the literature on whole body metabolism (Tattersall et al. 2012; Price et al. 2017; Cooper et al. 2020b; Broggi et al. 2022) and with human studies showing important long-lasting consequences of embryonic and perinatal conditions for adult phenotypic quality (Warner and Ozanne 2010; Tamashiro and Moran 2010). Adult zebra finches that had hatched from reproductive attempts that were subjected to a constant heat treatment from pre-natal right through to the independence from their parents, showed significantly lower Leak metabolism compared to birds exposed to the periodic treatment when exposed to 40 °C as adults. Lower oxygen consumption during Leak stage may be interpreted as evidence of adaptive programming to prevent heat stress at high temperatures, because this is the mitochondrial process responsible for heat production in endotherms (Rolfe and Brand 1996). However, following *the uncoupling to survive hypothesis*, elevated Leak activity has been linked to higher protection from oxidative stress (Brand 2000; Divakaruni and Brand 2011; Koch et al. 2021). Given that exposure to high temperatures has been shown to promote ROS production (Mujahid et al. 2005; Tan et al. 2010), lower metabolism of the Leak stage may instead represent a potential cost. Similarly, the trends we detected for Routine and OxCE, despite being non-significant, should also imply a lower risk of heat stress and thus may be an advantageous programming response of adaptive value as has been observed in king penguin’s *pectoralis* muscle mitochondria under cold conditions (Roussel et al. 2020).

However, it must be noted that in isolated cells the rate of Leak metabolism is slow compared to, for instance, routine oxygen consumption, as visible in Fig. 3. As a consequence, Routine can produce far more heat than Leak respiration. Therefore, to prevent heat stress at high temperature, it would be more efficient to decrease mitochondrial metabolism as a whole rather than just Leak respiration. In addition, when Leak respiration is altered, the thermogenic capacity of mitochondria changes only if its efficiency is also altered. In the present study, OxCE did not significantly change, suggesting that the reported decrease in Leak respiration may be better interpreted as an overall decrease in the oxidative activity of mitochondria rather than as a change in Leak per se. Interestingly, this overall decrease in the respiratory activity can still be considered an advantageous programming response to limit heat stress.

Our post hoc tests for the mitochondrial reserve capacity (FCR _R/ETS_) showed that the two early-life heat treatments did not differ. However, the interaction term was significant, and indeed the birds belonging to the constant experiment decreased, while the birds of the periodic experiment showed an increase in FCR _R/ETS_ (Fig. 3b; Appendix A, Table 12). This trend is similar to our findings for Leak stage (Fig. 3a; Appendix A, Table 4) and Routine (Fig. 3c; Appendix A, Table 2), but opposite to what we found for OxCE (Fig. 3d; Appendix A, Table 10). Interestingly, a decline in FCR _R/ETS_ is associated with an increase in damage to energy requiring tissues (Desler et al. 2012). These results suggest that the birds from the constant experiment may face a trade-off between a potentially beneficial reduction in Leak stage respiration as a response to heat, and a detrimental decrease in FCR_R/ETS_ leading to pathologies affecting tissues such as the heart and muscles (Sansbury et al. 2011), but this idea requires further testing.

In contrast to the constant heat treatment birds, the group of birds that experienced a more variable regime, with high temperatures only during the nestling stage, showed no interactions with the treatment in adulthood. This absence of carry-over effects deserves attention, since a previous study on these same birds had demonstrated increased mitochondrial Routine and Leak stages metabolism in individuals from heated nests almost three months after the treatment was ended, i.e. when the birds were a few months old (Ton et al. 2021). A similar increase was also identified in another recent study on zebra finches testing the role of acoustic cues during embryonic development in programming mitochondrial functions to heat challenges administered a couple of weeks after the end of the pre-natal treatment (Udino et al. 2021). Therefore, these two independent studies (Ton et al. 2021; Udino et al. 2021), both, revealed the effects of treatments early in development on mitochondrial function within a few months, but these effects were not found to persist further into adulthood in our present study. The lack of interactions between early life and adult treatment on a group of birds that had already shown adjustments in mitochondrial functions to heat at a younger age may indicate a weakening of mitochondrial changes over time, and or perhaps the weaker efficacy of this periodic early-life treatment. Indeed, differences in our long-term results reinforce the findings of previous studies suggesting that the presence of carry-over effects may depend on the intensity and timing of heat exposure (Loyau et al. 2015; Price et al. 2017). A good example of the role that the pattern of heat exposure has on mitochondrial functions is also shown by our results for Leak stage. Indeed, for the 40 °C treatment temperature in adulthood, the constant heated birds at 35 °C did not differ from the constant heated birds at 18 °C despite the 17 °C difference between the two groups, but they had lower oxygen consumption compared to both periodically heated groups of birds (Fig. 3a; Appendix A, Table 4). This suggests that differences in temperature variations between the treatments in early life may have stronger influences on mitochondrial functions compared to the differences between mean temperatures experienced.

The mitochondrial changes detected in the group of birds that were heated during the whole early life instead of only during the nestling stage, also suggest that the embryonic period may potentially represent a more suitable time for programming. Previous studies have shown that embryonic development is a temperature-sensitive stage leading to variation in mitochondrial function (Loyau et al. 2014, 2015; He et al. 2021; Stier et al. 2022). Instead, the post-natal stage may be more of a time for cell proliferation providing less flexibility for functional adjustments to changes in temperature, at least for altricial species (but see Nord and Giroud 2020). Identifying the most sensitive window to environmental conditions during growth and development may provide critical information for our understanding of carry-over effects, the relationship between abiotic conditions and physiological mechanisms, and conservation strategies.

The other important difference between our two experimental groups is with respect to the potential for indirect parental effects. In the constant treatment, the experimental individuals were produced in a heated environment in which their parents were also subjected to the experimental heat during the nesting and laying period, as well as the incubation and rearing period. By contrast, our periodic experimental treatment only heated the nest after the nestlings had hatched. It is, therefore, possible that the programming of mitochondrial function that we have demonstrated in this more comprehensively treated group may result from parental effects that were transmitted from the parents through egg provisioning (Lanni et al. 2016; Lassiter et al. 2018).

Our findings suggest that birds may adjust their physiology in benefit of their survival when exposed to constant higher ambient temperatures during development, but not when experiencing acute heat wave-like events in early-life stages. This variable response points to the potential complexity of responses in mitochondrial metabolism to heat, while also emphasising how easily different experimental approaches can lead to opposing conclusions. The periodic heat treatment (during the nestling stage only) was quite effective in eliciting a functional mitochondrial response a few months after the treatment (Ton et al. 2021) but did not lead to a more sustained effect. Had we not contrasted that periodic treatment, with the constant heat treatment (which did elicit a response after 2 years) we may have concluded that early-life exposure does not produce sustained effects on mitochondrial function much later in life. Therefore, the contrast in the short- and long-term outcomes of the different experimental treatments that we have found indicates the complexity around mitochondrial responses to early-life experience, or potentially parental effects.

In addition to further exploring this complexity and trying to identify the mechanisms and limits of mitochondrial programming, further study is required to test the ecological and physiological relevance of reduced Leak metabolism (Koch et al. 2021). An important question is the extent to which the changes that we have observed in mitochondrial function are adaptive, and through what pathways. For example, are changes to Leak components of mitochondrial respiration adaptive in helping individuals to reduce body temperature in hot ambient conditions, or do they reduce oxidative stress? Alternatively, it is of course possible that the observed differences are not adaptive, and are produced as an outcome of altered development, with little physiological impact on performance or evolutionary fitness. Indeed, whilst the idea of adaptive programming is ecologically attractive, zebra finches in the wild experience highly variable thermal environments, both during development, but also throughout their lifetime (Griffith et al. 2020). A long-sustained response to heat during early life may in fact impede the ability of individuals to tolerate the variety of conditions that they will encounter throughout their lifetime. In this context, the more immediate and consistent acclimation response that we observed across all the birds to the experimental heatwave makes more sense. Further study is required to evaluate the relative importance of immediate and long-term responses to heatwaves, as well as evaluating what implications such responses might have for individual fitness.

## Supplementary Information

Below is the link to the electronic supplementary material.

Supplementary file1 (PDF 1681 KB)

Supplementary file2 (PDF 957 KB)

## Data Availability

The datasets generated during the current study are publicly available at The Open Science Framework data repository. (DP170103619).
