# Supplementary material for: Short- and long-term consequences of heat exposure on mitochondrial metabolism in zebra finches (Taeniopygia castanotis)
Source: Oecologia. 2023 Mar 10;201(3):637–48. doi: 10.1007/s00442-023-05344-7 (PMC10038956; doi:10.1007/s00442-023-05344-7)
Supplement: Supplementary file 1 — Supplementary file1 (PDF 1681 KB) [file 442_2023_5344_MOESM1_ESM.pdf]

Short and long-term consequences of heat exposure on mitochondrial metabolism in zebra finches (*Taeniopygia castanotis*)

Supplementary Material

Contents

1 Introduction **2**

2 Descriptive Statistics **3**
2.1 Data description ... 3
2.1.1 Experiments in Early Life Stages ... 3
2.1.2 Sex ... 4
2.2 Observations ... 4
2.2.1 Number of Observations ... 4
2.2.2 Predictors ... 4
2.3 Outcomes ... 5

3 Evaluating Early Life Control **7**
3.1 Routine ... 7
3.2 Leak ... 8
3.3 OxPhos ... 9
3.4 ETS ... 9
3.5 OxCE ... 10
3.6 FCRs ... 11
3.7 Discussion ... 12

4 Statistical Approach **13**
4.1 Model Comparison ... 13
4.2 Estimation details ... 13

5 Routine Analysis **14**
5.1 Model comparison ... 14
5.2 Selected Model ... 14
5.2.1 Fit and Checks ... 14
5.2.2 Effects ... 16

6 Leak Analysis **22**
6.1 Model comparison ... 22
6.2 Selected Model ... 22
6.2.1 Fit and Checks ... 22
6.2.2 Effects ... 24

7 OxPhos Analysis **29**
7.1 Model comparison ... 29

7.2 Selected Model ... 29
7.2.1 Fit and Checks ... 29
7.2.2 Effects ... 31

8 ETS Analysis **35**
8.1 Model comparison ... 35
8.2 Selected Model ... 35
8.2.1 Fit and Checks ... 35
8.2.2 Effects ... 37

9 OxCE Analysis **41**
9.1 Model comparison ... 41
9.2 Selected Model ... 41
9.2.1 Fit and Checks ... 41
9.2.2 Effects ... 43

10 FCR_5 Analysis **48**
10.1 Model comparison ... 48
10.2 Selected Model ... 48
10.2.1 Fit and Checks ... 48
10.2.2 Effects ... 50

11 Extra **55**

References **56**

1 Introduction

In this report, we present the details of the statistical analyses for the article *"Short and long-term consequences of heat exposure on mitochondrial metabolism in zebra finches (Taeniopygia castanotis)"*.

The aim of the study is to test variation in mitochondrial metabolism at different temperatures, and the presence of temporally stable carry-over effects of early heat stress into adulthood by exposing zebra finches to a series of thermal manipulations.

The Supplemental Material is organized as follows:

- Section 2 Descriptive Statistics
- Section 3 Evaluating Early Life Control
- Section 4 Statistical Approach
- Section 5 Routine Analysis
- Section 6 Leak Analysis
- Section 7 OxPhos Analysis
- Section 8 ETS Analysis
- Section 9 OxCE Analysis
- Section 10 FCR_s Analysis

2 Descriptive Statistics

The data includes 235 on 65 birds. In particular, we consider the following variables:

- **Nest_ID** - factor indicating the nest unique ID (33 levels).
- **Band_ID** - factor indicating the bird unique ID (65 levels).
- **Sex** - factor indicating the bird sex. Levels are: `"Female"` and `"Male"`.
- **Early_treat** - factor indicating the bird early treatment condition. Levels are: `"Control 18C"`, `"Control 21C"`, `"Constant 35C"`, and `"Periodic 40C"`.
- **Adult_treat** - factor indicating the bird adult treatment condition. Levels are: `"Control 25C"` and `"Treat 40C"`.
- **Proteincont** - numeric value indicating the sample protein content.
- **Routine** - numeric value indicating the basal mitochondrial oxygen consumption (pmol O_2 s^{-1} mL^{-1}).
- **Leak** - numeric value indicating the mitochondrial oxygen consumption related to proton leak (pmol O_2 s^{-1} mL^{-1}).
- **OxPhos** - numeric value indicating the oxidative phosphorylation (computed as `Routine - Leak`; pmol O_2 s^{-1} mL^{-1}).
- **ETS** - numeric value indicating the mitochondrial oxygen consumption during electron transport system (pmol O_2 s^{-1} mL^{-1}).
- **OxCE** - numeric value indicating the OxPhos coupling efficiency (computed as `1 - (Leak / Routine)`).
- **FCR** - numeric value indicating the mitochondrial reserve capacity (computed as `Routine/ ETS`).

2.1 Data description

2.1.1 Experiments in Early Life Stages

Birds used in the present experiment were exposed to different thermal manipulations during the early stages of their lives. In particular, birds were included in two different experiments we refer to as `"Constant"` and `"Periodic"`.

In the `"Constant"` experiment, the control group was kept at a *constant* temperature of 18°C, whereas the treatment group was kept at a *constant* temperature of 35°C.

In the `"Periodic"` experiment, the control group was kept at a temperature of 21.5°C (±1.84°C), whereas the treatment group was kept at a *periodic* temperature of 40°C for six hours a day.

The number of birds according to experiment and group in the early life stages is presented in Table 1.

Table 1: Number of birds according to experiment and group in the early life stages

	Group		
Early Experiment	Control	Treat	Total
Constant	20	12	32
Periodic	19	14	33

2.1.2 Sex

The number of birds according to experiment and group in the early life stages is presented in Table 2.

Table 2: Sex according to experiment and group in the early life stages

	Constant Early Treatment		Periodic Early Treatment		
Sex	Constant 18°C	Constant 35°C	Periodic 21°C	Periodic 40°C	Total
Female	9	6	7	6	28
Male	11	13	5	8	37

2.2 Observations

2.2.1 Number of Observations

The number of observations in the current experiment according to the birds' past experiments in the early life stages, is presented in Table 3.

Table 3: Number of observations according to adult and early treatment condition

	Constant Early Treatment		Periodic Early Treatment		
Adult Treatment	Constant 18°C	Constant 35°C	Periodic 21°C	Periodic 40°C	Total
Control 25C	35	24	36	28	123
Treat 40C	34	23	30	25	112

2.2.2 Predictors

Summary information of the quantitative predictors are reported in Table 4 and distributions are presented in Figure 1.

Table 4: Summary infromation of quantitative predictors

Predictor	n	Min.	1st Qu.	Median	Mean	3rd Qu.	Max.	sd
Protein Content	235	2.75	4.24	4.9	4.96	5.59	8.82	1.05

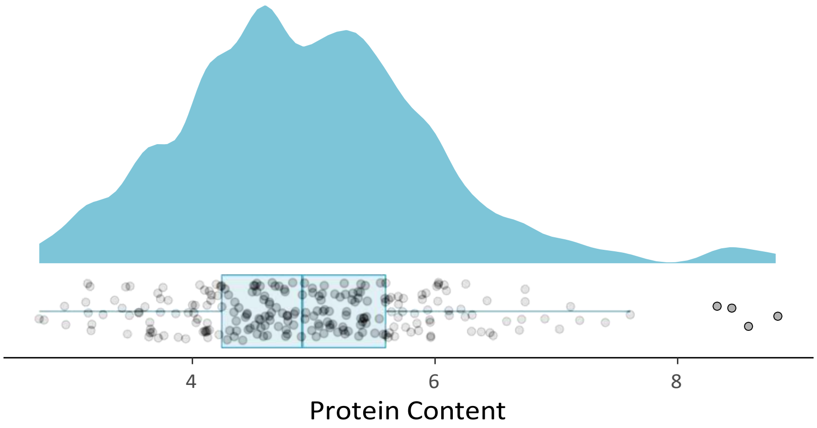

Figure 1: Distributions of the quantitative predictors

2.3 Outcomes

Summary information of the quantitative outcomes are reported in Table 5 and distributions are presented in Figure 2.

Table 5: Summary infromation of quantitative outcomes

Outcome	n	Min.	1st Qu.	Median	Mean	3rd Qu.	Max.	sd
Routine	235	13.89	23.23	26.49	26.53	29.90	41.92	4.90
ETS	235	20.77	71.60	79.70	77.78	87.47	114.37	15.24
Leak	235	2.22	5.97	7.42	7.38	8.74	12.26	1.90
OxPhos	235	10.53	16.70	18.67	19.15	21.71	31.24	3.78
OxCE	235	0.53	0.69	0.72	0.72	0.76	0.88	0.05
FCR	235	0.20	0.30	0.33	0.36	0.37	1.17	0.12

Note: Routine, ETS, Leak, and OxPhos unit measure pmol O_2 s^{-1} mL^{-1}

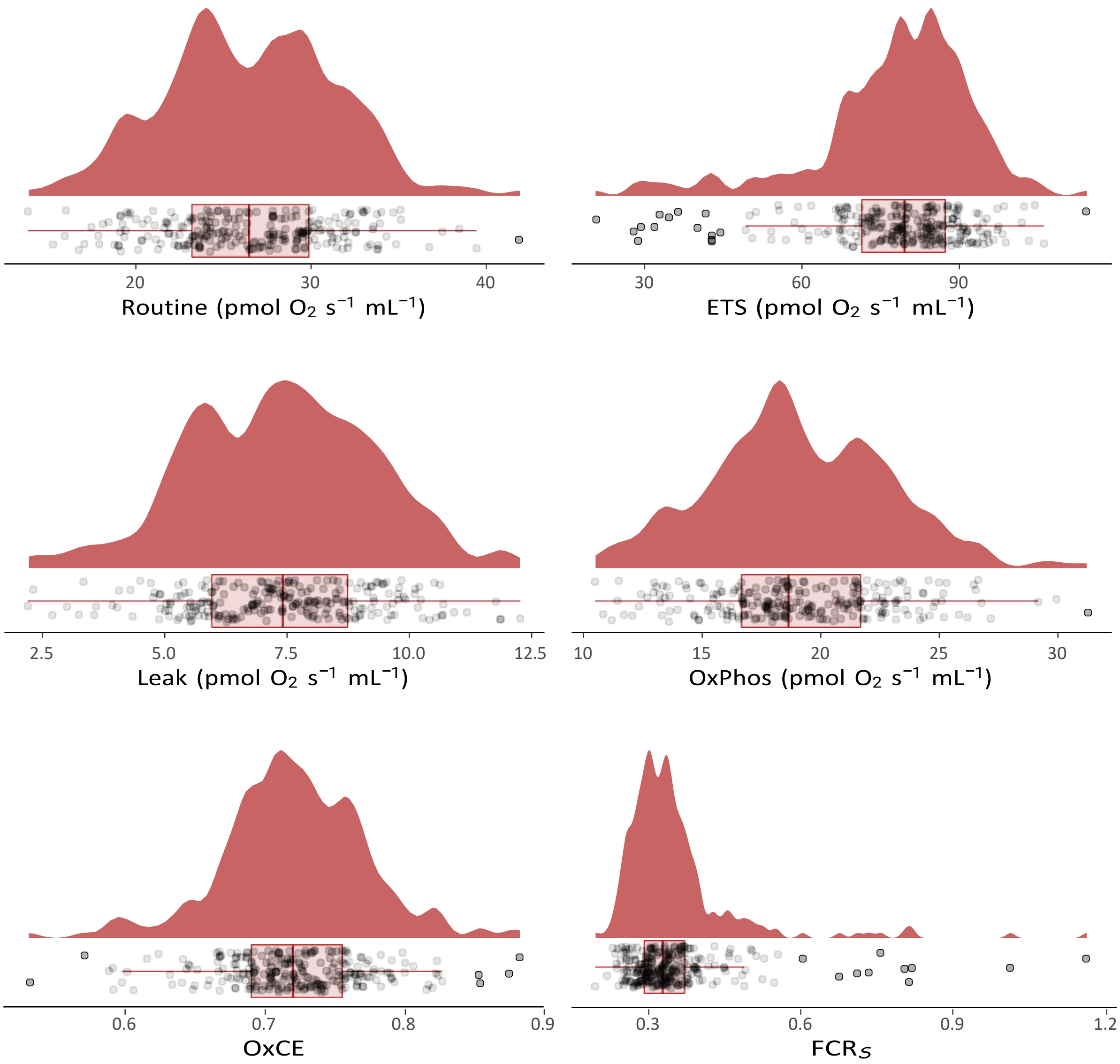

Figure 2: Distributions of the quantitative outcomes

3 Evaluating Early Life Control

In this section, we evaluate if there is any mitochondrial metabolism difference between the two control groups of the early life experiments.

We fit a Mixed effect model for each outcome of interest considering as random factor the `Band_ID` and as fixed effects the `Proteincont_cent` (centred), `Sex` and the interaction between `Adult_treat` and `Early_treat`.

If there is no difference we expect neither the interaction between `Adult_treat` and `Early_treat` nor the single effect of `Early_treat` to be significant.

3.1 Routine

```
early_routine <- lmer(Routine ~ Proteincont_cent + Sex + Early_treat * Adult_treat + (1|Band_ID),
                      data = data_early, REML = TRUE)
```

Table 6: Analysis of Deviance Table (Type II Wald F tests with Kenward-Roger df)

Effects	F	Df	Df.res	Pr(>F)	
Proteincont_cent	34.89	1	128.93	3e-08	***
Sex	2.86	1	37.17	0.10	.
Early_treat	5.39	1	35.40	0.03	*
Adult_treat	1.90	1	105.33	0.17	
Early_treat:Adult_treat	0.06	1	99.26	0.81	

Note: 0 '***' 0.001 '**' 0.01 '*' 0.05 '.' 0.1 ' ' 1

There is a statistically significant effect of `Early_treat`. The difference between the two control groups is presented in Figure 3.

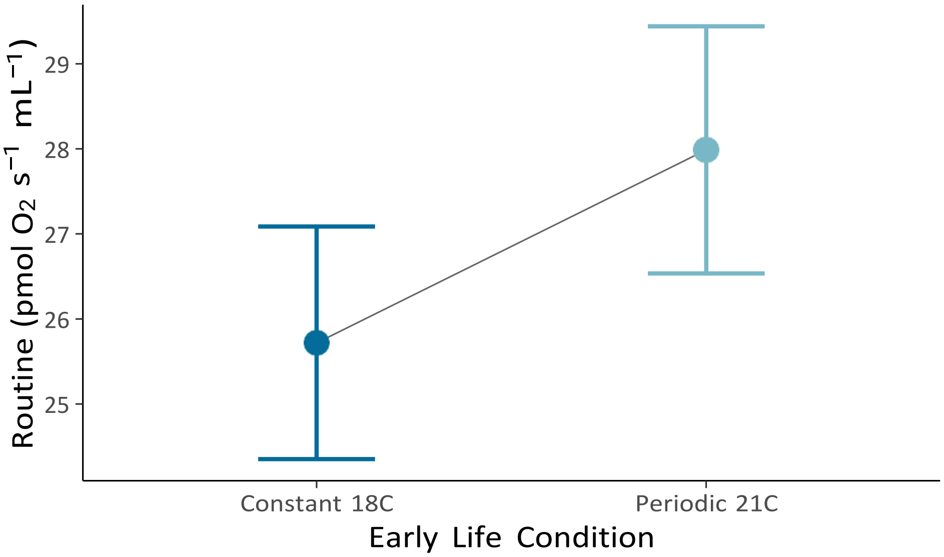

Figure 3: Difference between the two control groups

3.2 Leak

```
early_leak <- lmer(Leak ~ Proteincont_cent + Sex + Early_treat * Adult_treat + (1|Band_ID),
                   data = data_early, REML = TRUE)
```

Table 7: Analysis of Deviance Table (Type II Wald F tests with Kenward-Roger df)

Effects	F	Df	Df.res	Pr(>F)	
Proteincont_cent	22.26	1	128.99	6e-06	***
Sex	5.80	1	37.17	0.021	*
Early_treat	12.09	1	35.42	0.001	**
Adult_treat	0.03	1	105.11	0.864	
Early_treat:Adult_treat	0.94	1	99.12	0.334	

Note: 0 '***' 0.001 '**' 0.01 '*' 0.05 '.' 0.1 ' ' 1

There is a statistically significant effect of `Early_treat`. The difference between the two control groups is presented in Figure 4.

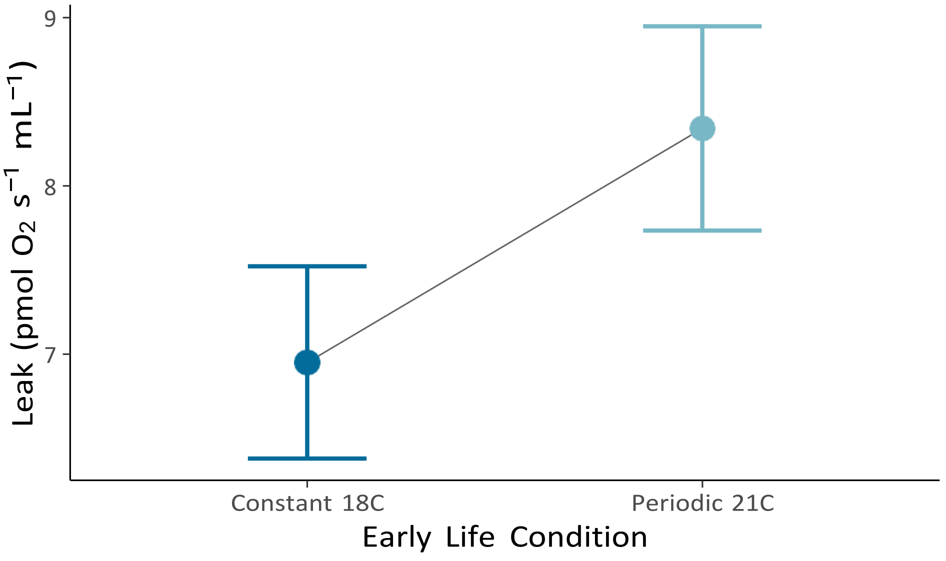

Figure 4: Difference between the two control groups

3.3 OxPhos

```
early_oxphos <- lmer(OxPhos ~ Proteincont_cent + Sex + Early_treat * Adult_treat + (1|Band_ID),
                     data = data_early, REML = TRUE)
```

Table 8: Analysis of Deviance Table (Type II Wald F tests with Kenward-Roger df)

Effects	F	Df	Df.res	Pr(>F)	
Proteincont_cent	28.05	1	128.40	5e-07	***
Sex	0.77	1	37.15	0.38	
Early_treat	1.08	1	35.53	0.31	
Adult_treat	2.99	1	103.88	0.09	.
Early_treat:Adult_treat	0.67	1	98.33	0.42	

Note: 0 '***' 0.001 '**' 0.01 '*' 0.05 '.' 0.1 ' ' 1

There is no statistically significant effect of `Early_treat`.

3.4 ETS

```
early_ets <- lmer(ETS ~ Proteincont_cent + Sex + Early_treat * Adult_treat + (1|Band_ID),
                  data = data_early, REML = TRUE)
```

Table 9: Analysis of Deviance Table (Type II Wald F tests with Kenward-Roger df)

Effects	F	Df	Df.res	Pr(>F)	
Proteincont_cent	3.79	1	128.09	0.05	.
Sex	2.94	1	37.16	0.09	.
Early_treat	1.15	1	35.31	0.29	
Adult_treat	1.43	1	106.23	0.23	
Early_treat:Adult_treat	2.60	1	99.89	0.11	

Note: 0 ‘***’ 0.001 ‘**’ 0.01 ‘*’ 0.05 ‘.’ 0.1 ‘ ’ 1

There is no statistically significant effect of `Early_treat`.

3.5 OxCE

In this case, `Proteincont_cent` is not included.

```
early_oxce <- lmer(OxCE ~ Sex + Early_treat * Adult_treat + (1|Band_ID),
                   data = data_early, REML = TRUE)
```

Table 10: Analysis of Deviance Table (Type II Wald F tests with Kenward-Roger df)

Effects	F	Df	Df.res	Pr(>F)	
Sex	3.05	1	36.22	0.09	.
Early_treat	4.85	1	35.97	0.03	*
Adult_treat	1.78	1	98.09	0.19	
Early_treat:Adult_treat	2.12	1	98.19	0.15	

Note: 0 ‘***’ 0.001 ‘**’ 0.01 ‘*’ 0.05 ‘.’ 0.1 ‘ ’ 1

There is a statistically significant effect of `Early_treat`. The difference between the two control groups is presented in Figure 5.

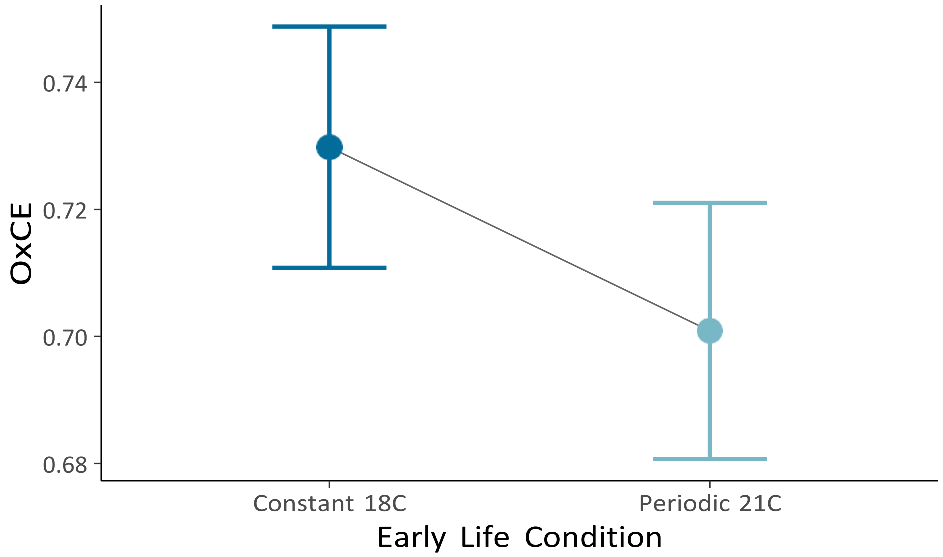

Figure 5: Difference between the two control groups

3.6 FCRs

In this case, `Proteincont_cent` is not included.

```
early_fcr <- lmer(FCR ~ Sex + Early_treat * Adult_treat + (1|Band_ID),
                  data = data_early, REML = TRUE)
```

Table 11: Analysis of Deviance Table (Type II Wald F tests with Kenward-Roger df)

Effects	F	Df	Df.res	Pr(>F)	
Sex	0.72	1	36.08	0.402	
Early_treat	0.56	1	35.37	0.458	
Adult_treat	0.05	1	102.31	0.820	
Early_treat:Adult_treat	7.44	1	102.52	0.008	**

Note: 0 '***' 0.001 '**' 0.01 '*' 0.05 '.' 0.1 ' ' 1

There is a statistically significant interaction between `Early_treat` and `Adult_treat`. The difference between the two control groups is presented in Figure 6.

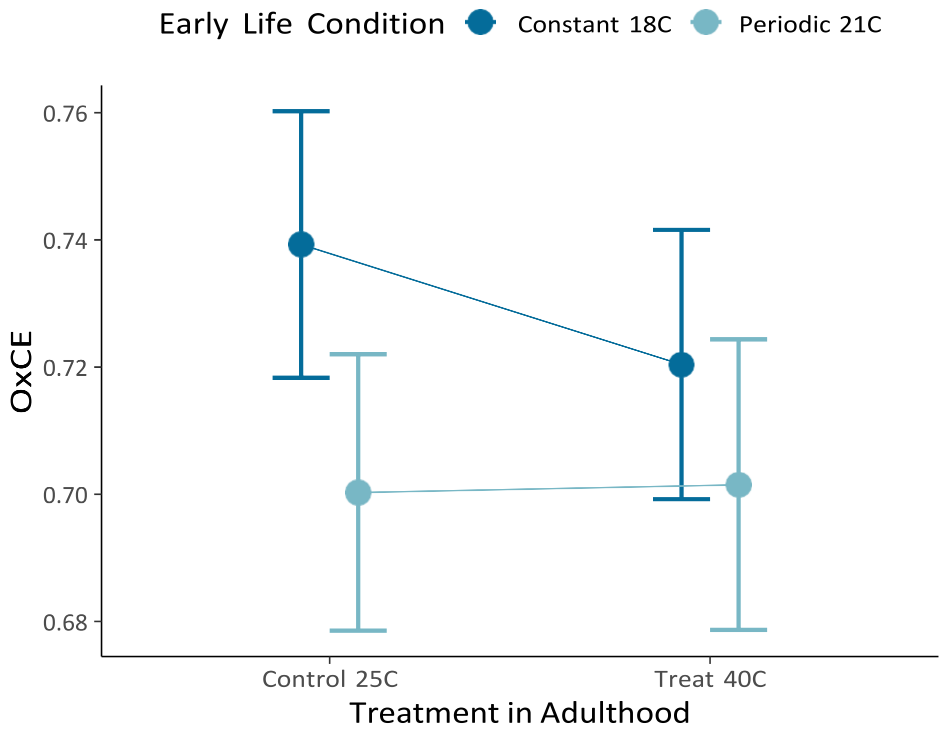

Figure 6: Difference between the two control groups

3.7 Discussion

Results indicate a statistically significant difference for Routine, Leak, OxCE, and FCR_S in the two control groups.

Given the results, in the following analysis, we maintain the two control groups separate.

4 Statistical Approach

Statistical analyses are conducted separately for each outcome of interest (i.e., Routine, Leak, OsPhos, ETS, OxCE, FCR_s).

Analyses were carried out using R software (v4.1.2 R Core Team, 2021).

4.1 Model Comparison

Model comparison involves selecting the most plausible statistical model given the data and a set of candidate models (McElreath, 2020).

During the process of model comparison, first, the research hypotheses must be formalized as statistical models. According to the predictors included in the model, researchers define the variables expected to have an important role in the phenomenon of interest. Subsequently, the obtained models are compared in terms of the statistical evidence (i.e., support by the data) using information criteria (Wagenmakers & Farrell, 2004).

Information criteria provide an estimate of the average deviance (i.e., error) of a model's ability to predict new data, and thus lower values are interpreted as evidence of a better model (McElreath, 2020). This allows us to consider the trade-off between parsimony and goodness-of-fit (Vandekerckhove et al., 2015) when evaluating models; as the complexity of a model increases(i.e., more parameters), its fit to the data increases, but generalizability (i.e., ability to predict new data) decreases. The researchers aim to find the right balance between fit and generalizability to describe, with a statistical model, the important features of the studied phenomenon, but not the random noise of the observed data.

Model comparison favors models with effects that offer an appropriate description of the data generating process, penalizing the inclusion of further, unnecessary effects that may increase redundancies and complexity. The Akaike information criterion (AIC; Akaike, 1973) is used to select the most plausible among the considered models, given the data.

4.2 Estimation details

We report some details about model estimation.

We follow good practice of centering continuous predictors (i.e., `Proteincont`).

We considered model m0 as a reference model to evaluate the possible contribution of the other variables. In model m1, we included early life experiments. In model m2, we included treatment in adulthood. In model m3, we include both early life and adulthood treatments to evaluate their additive effect. Finally, in model m4, we include the interaction between early life and adulthood treatments to evaluate carry over effects of early life experiments in adulthood.

Using R model syntax, the models are:

- m_0: outcome ~protein content + sex + (1|band)
- m_1: outcome ~early life experiments + protein content + sex + (1|band)
- m_2: outcome ~treatments in adulthood + protein content + sex + (1|band)
- m_3: outcome ~ treatments in adulthood + early life experiments + protein content + sex + (1|band)
- m_4: outcome ~ treatments in adulthood * early life experiments + protein content + sex + (1|band)

Model weights were computed for AIC indicating the probability that each model would make the best predictions for new data, conditional on the set of models considered (McElreath, 2020). With this method, models are compared using a continuous informative measure of evidence, rather than a dichotomous decision.

For each model, we report in the present article and in the supplemental materials:

- Model summary with information about the estimated model parameters
- R2 fit index computed using the MuMIn R-package (Bartoń, 2019)
- Model assumptions checks using the performance R-package (Lüdecke et al., 2021)
- Predictors statistical significance test (i.e. Wald chisquare tests) using the car R-package (Fox, 2015).
- Predicted values and post-hoc tests using the emmeans R-package (Lenth, 2021).

Finally, Mixed Models can be estimated using the Maximum Likelihood (ML) or the Restricted Maximum Likelihood (REML) approach. REML allows to obtain more precise estimates, but can not be used when comparing models with different fixed effects (Fox, 2015). Therefore, we estimated models using the ML approach in the model comparison and subsequently we re-estimate the selected models using the REML approach.

5 Routine Analysis

5.1 Model comparison

Results of the model comparison are reported in Table 12.

Table 12: Model comparison using AIC and BIC.

Model	Term	Df	AIC	$AIC_{weights}$
m_0		5	1349.83	2%
m_1	Early_treat	8	1347.18	6%
m_2	Adult_treat	6	1346.42	9%
m_3	Early_treat + Adult_treat	9	1343.77	32%
m_4	Early_treat * Adult_treat	12	1342.85	51%

Considering the AIC weights, model `m_4` (the model with the interaction `Early_treat * Adult_treat`) is the best model (51%) We can interpret these results as there is some evidence in favor of the interaction but we can not draw strong conclusions.

In the following analysis, we consider model `m_4`.

5.2 Selected Model

We re-estimate the selected model using the REML approach.

```
model_routine <- lmer(Routine ~ Early_treat * Adult_treat + Sex + Proteincont_cent + (1|Band_ID),
                      data = data_mito, REML = TRUE)
```

5.2.1 Fit and Checks

The summary of the model is presented in Table 13.

Table 13: Model summary

Term	Estimate	Std. Error	2.5 %	97.5 %	df	t value	Pr(>|t|)	
**Random Effets (sd)**								
Band_ID	2.26		1.41	2.86				
Residual	3.63		3.23	4.00				
**Fixed Effets**								
(Intercept)	27.33	0.90	25.62	29.05	95.55	30.29	<2e-16	***
Proteincont_cent	2.19	0.29	1.60	2.75	216.18	7.44	2e-12	***
SexMale	-2.07	0.77	-3.54	-0.61	58.87	-2.70	0.009	**
Early_treatPeriodic 21C	2.20	1.14	0.03	4.37	107.40	1.93	0.056	.
Early_treatConstant 35C	0.74	1.28	-1.70	3.17	103.05	0.58	0.564	
Early_treatPeriodic 40C	0.67	1.21	-1.64	2.99	103.52	0.55	0.580	
Adult_treatTreat 40C	-0.95	0.93	-2.79	0.83	180.90	-1.02	0.307	
Early_treatPeriodic 21C:Adult_treatTreat 40C	0.20	1.29	-2.32	2.70	175.07	0.15	0.877	
Early_treatConstant 35C:Adult_treatTreat 40C	-2.47	1.41	-5.19	0.29	171.69	-1.76	0.081	.
Early_treatPeriodic 40C:Adult_treatTreat 40C	1.29	1.35	-1.31	3.93	171.91	0.96	0.340	

Note: 0 '***' 0.001 '**' 0.01 '*' 0.05 '.' 0.1 ' ' 1

The fit of the model is presented in Table 14.

Table 14: Model fit

Marginal R^2	Conditional R^2
29.3%	49.1%

Model assumptions checks are presented in Figure 7.

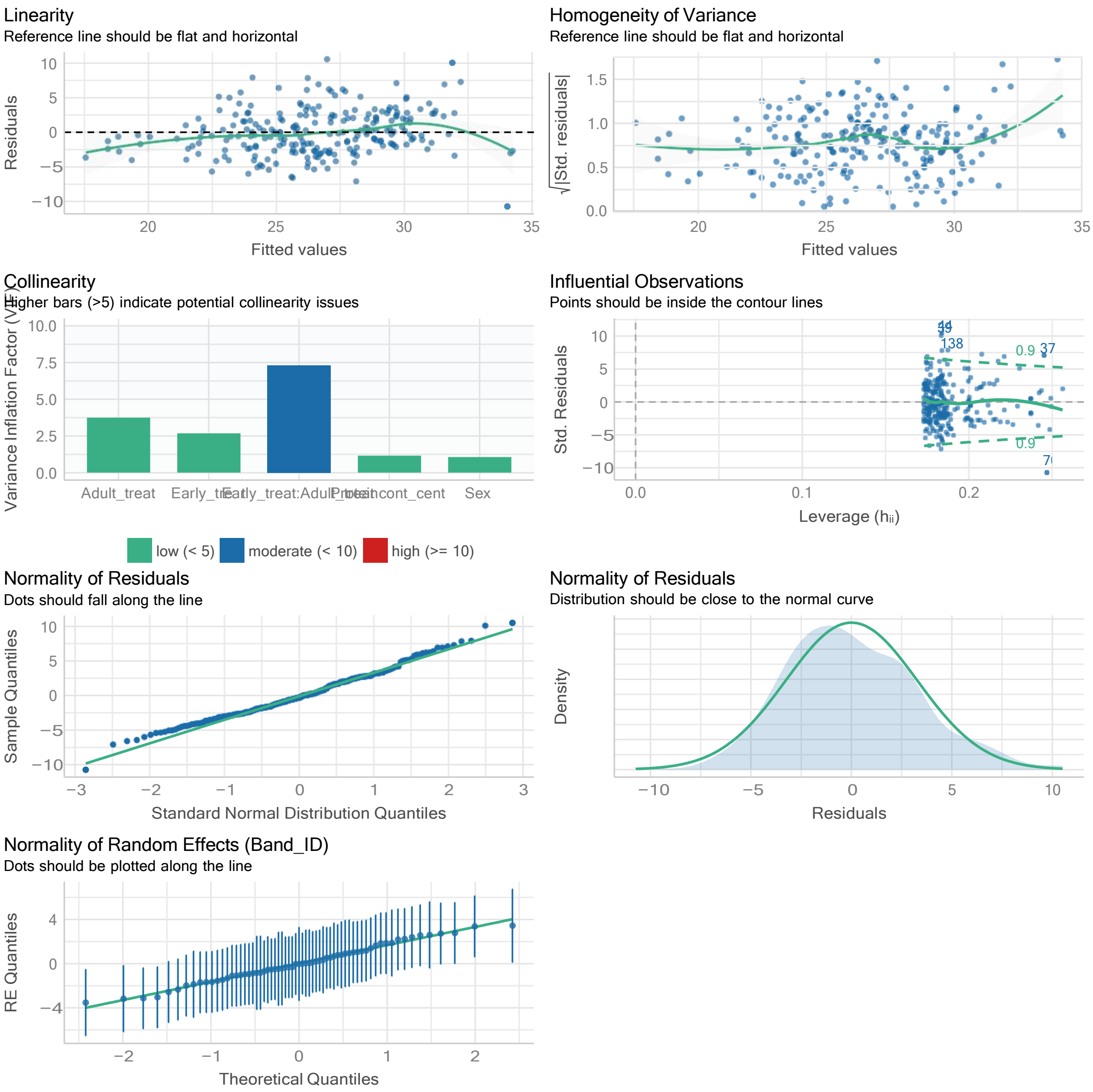

Figure 7: Model assumptions checks

Overall, the model behaves well and the fit to the data is good.

5.2.2 Effects

To evaluate predictors' statistical significance, we consider Wald F tests. Results are reported in Table 15.

Table 15: Analysis of Deviance Table (Type II Wald F tests with Kenward-Roger df)

Effects	F	Df	Df.res	Pr(>F)	
Proteincont_cent	54.15	1	216.56	4e-12	***
Sex	7.26	1	60.85	0.009	**
Early_treat	2.79	3	58.24	0.048	*
Adult_treat	5.30	1	180.47	0.022	*
Early_treat:Adult_treat	2.31	3	173.40	0.078	.

Note: 0 '***' 0.001 '**' 0.01 '*' 0.05 '.' 0.1 ' ' 1

Note that the interaction `Adult_treat:Early_treat` is not statistically significant but close to the statistical threshold. This is in line with the model comparison results that indicated some evidence in favor of the interaction but no strong conclusions can be drawn.

We evaluate the effect by presenting the predicted values and post-hoc tests.

- **Protein Content.** Estimated effect is reported in Table 16 and presented in Figure 8.

Table 16: Estimated effects

Term	Estimate	Std. Error	2.5 %	97.5 %	df	t value	Pr(>|t|)	
Proteincont_cent	2.19	0.29	1.6	2.75	216.18	7.44	2e-12	***

Note: 0 '***' 0.001 '**' 0.01 '*' 0.05 '.' 0.1 ' ' 1

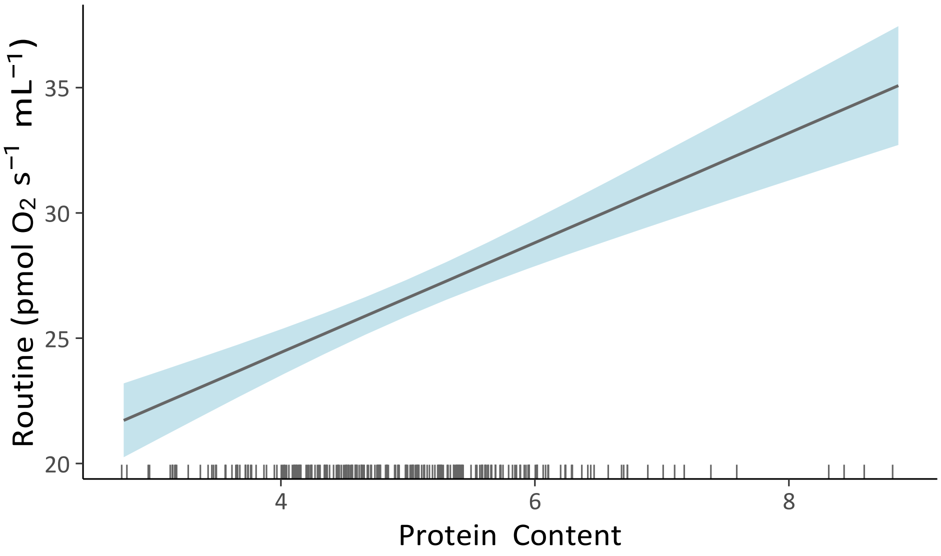

Figure 8: Protein Content effect

- **Sex.** Predicted values are reported in Table 17 and presented in Figure 9. Post hoc tests are reported in Table 18.

```
emmeans_sex <- emmeans::emmeans(model_routine, pairwise ~ Sex, adjust = "mvt")
```

Table 17: Predicted values

Sex	Predicted	SE	df	2.5%	97.5%
Female	27.64	0.57	60.43	26.50	28.78
Male	25.57	0.51	58.26	24.55	26.58

Table 18: Post hoc contrasts

Contrast	Estimate	SE	df	t.ratio	p.value	
Female - Male	2.07	0.77	60.85	2.69	0.009	**

Note: 0 '***' 0.001 '**' 0.01 '*' 0.05 '.' 0.1 ' ' 1

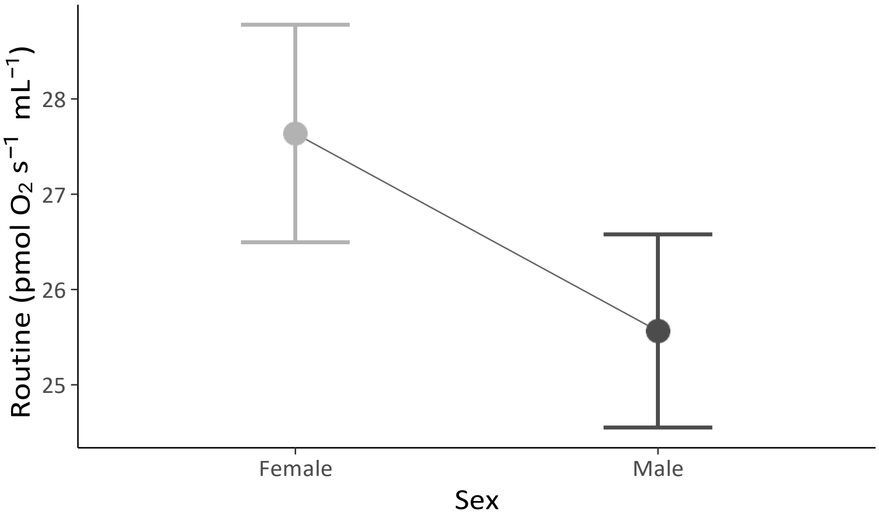

Figure 9: Sex predicted values

- **Adult and Early Treatment Interaction.** Predicted values are reported in Table 19 and presented in Figure 10. Post hoc tests are reported in Table 20.

```
emmeans_int <- emmeans::emmeans(model_routine, ~ Early_treat*Adult_treat)
```

Table 19: Predicted values

Early Life Condition	Treatment in Adulthood	Predicted	SE	df	2.5%	97.5%
Constant 18C	Control 25C	26.30	0.81	114.82	24.70	27.90
Periodic 21C	Control 25C	28.49	0.81	104.09	26.88	30.10
Constant 35C	Control 25C	27.04	0.99	100.33	25.07	29.00
Periodic 40C	Control 25C	26.97	0.92	101.36	25.14	28.80
Constant 18C	Treat 40C	25.35	0.83	120.82	23.70	26.99
Periodic 21C	Treat 40C	27.74	0.87	121.68	26.01	29.47
Constant 35C	Treat 40C	23.61	1.00	104.31	21.62	25.60
Periodic 40C	Treat 40C	27.31	0.96	110.96	25.41	29.21

Table 20: Post hoc contrasts

Contrast	Estimate	SE	df	t.ratio	p.value[1]	
**Contrasts in the Adult Control 25C**						
Early 18C - 21C	-2.20	1.14	109.94	-1.93	0.49	
Early 18C - 35C	-0.74	1.28	105.58	-0.58	1.00	
Early 18C - 40C	-0.67	1.22	106.05	-0.55	1.00	
Early 21C - 35C	1.46	1.29	101.25	1.13	0.94	
Early 21C - 40C	1.52	1.22	103.09	1.25	0.90	
Early 35C - 40C	0.07	1.35	100.53	0.05	1.00	
**Contrasts in the Adult Treat 40C**						
Early 18C - 21C	-2.40	1.19	121.29	-2.01	0.44	
Early 18C - 35C	1.73	1.31	110.43	1.33	0.86	
Early 18C - 40C	-1.96	1.26	113.74	-1.56	0.74	
Early 21C - 35C	4.13	1.34	110.69	3.09	0.04	*
Early 21C - 40C	0.43	1.29	116.19	0.34	1.00	
Early 35C - 40C	-3.70	1.39	107.10	-2.66	0.12	
**Within Group Differences[2]**						
Early 18C	-0.95	0.93	182.48	-1.02	0.96	
Early 21C	-0.75	0.92	179.27	-0.82	0.99	
Early 35C	-3.42	1.06	166.56	-3.23	0.03	*
Early 40C	0.34	1.02	172.88	0.33	1.00	
**Differences Between Within Group Differences[3]**						
Early 18C - 21C	-0.20	1.29	176.80	-0.15	1.00	
Early 18C - 35C	2.47	1.41	173.51	1.76	0.61	
Early 18C - 40C	-1.29	1.35	173.72	-0.96	0.97	
Early 21C - 35C	2.67	1.40	172.00	1.91	0.50	
Early 21C - 40C	-1.09	1.36	173.96	-0.80	0.99	
Early 35C - 40C	-3.76	1.47	169.59	-2.56	0.15	

Note:

0 ’***’ 0.001 ’**’ 0.01 ’*’ 0.05 ’.’ 0.1 ’ ’ 1

[1] P value adjustment: mvt method for 22 tests

[2] Differences (Adult Treat 40C - Adult Control 25C) for each Early Life Condition

[3] Differences between slopes of each Early Life Condition

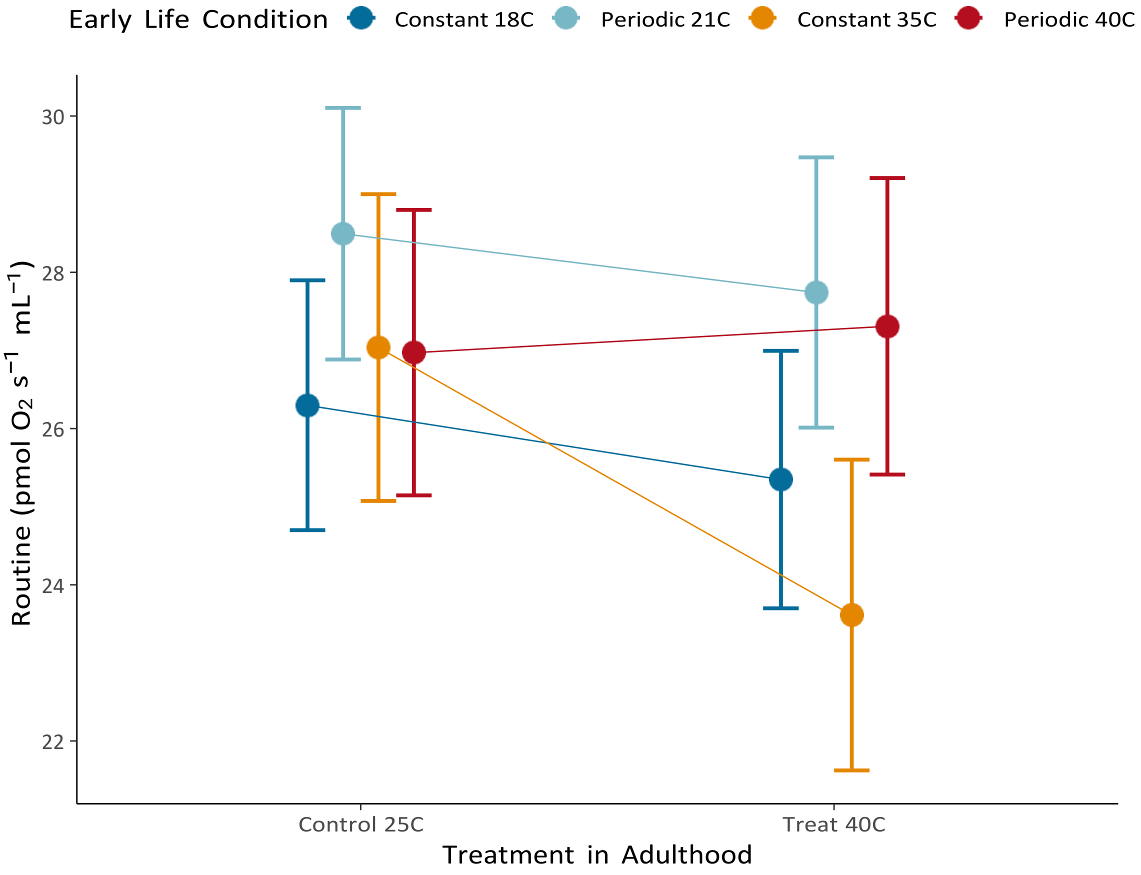

Figure 10: Interaction predicted values

6 Leak Analysis

6.1 Model comparison

Results of the model comparison are reported below,

Table 21: Model comparison using AIC and BIC.

Model	Term	Df	AIC	$AIC_{weights}$	
m_0		5	910.47	0%	
m_1	Early_treat	8	896.99	11%	
m_2	Adult_treat	6	912.32	0%	
m_3	Early_treat + Adult_treat	9	898.88	4%	
m_4	Early_treat * Adult_treat	12	892.86	85%	

Considering the AIC weights, model `m_4` (the model with the `Early_treat*Adult_treat` interaction) is the best model (85%) Therefore, we can say that there is some evidence in favor of the interaction between treatments at different life stages.

In the following analysis, we consider model `m_4`.

6.2 Selected Model

We re-estimate the selected model using the REML approach.

```
model_leak <- lmer(Leak ~ Early_treat*Adult_treat + Sex + Proteincont_cent + (1|Band_ID),
                   data = data_mito, REML = TRUE)
```

6.2.1 Fit and Checks

The summary of the model is presented in Table 22.

Table 22: Model summary

Term	Estimate	Std. Error	2.5 %	97.5 %	df	t value	Pr(>|t|)	
**Random Effets (sd)**								
Band_ID	0.84		0.53	1.05				
Residual	1.40		1.25	1.54				
**Fixed Effets**								
(Intercept)	7.37	0.34	6.71	8.02	104.11	21.54	<2e-16	***
Proteincont_cent	0.71	0.11	0.49	0.93	214.90	6.32	1e-09	***
SexMale	-1.09	0.29	-1.65	-0.54	64.12	-3.78	3e-04	***
Early_treatPeriodic 21C	1.66	0.43	0.83	2.48	116.66	3.83	2e-04	***
Early_treatConstant 35C	0.51	0.48	-0.42	1.43	112.15	1.05	0.297	
Early_treatPeriodic 40C	0.41	0.46	-0.47	1.29	112.67	0.89	0.374	
Adult_treatTreat 40C	0.32	0.36	-0.37	1.02	185.40	0.89	0.372	
Early_treatPeriodic 21C:Adult_treatTreat 40C	-0.53	0.50	-1.50	0.44	179.92	-1.05	0.294	
Early_treatConstant 35C:Adult_treatTreat 40C	-1.47	0.54	-2.52	-0.41	176.60	-2.70	0.008	**
Early_treatPeriodic 40C:Adult_treatTreat 40C	0.40	0.52	-0.62	1.40	176.84	0.76	0.448	

Note: 0 ‘***’ 0.001 ‘**’ 0.01 ‘*’ 0.05 ‘.’ 0.1 ‘ ’ 1

The fit of the model is presented in Table 23.

Table 23: Model fit

Marginal R^2	Conditional R^2
28.9%	47.6%

Model assumptions checks are presented in Figure 11.

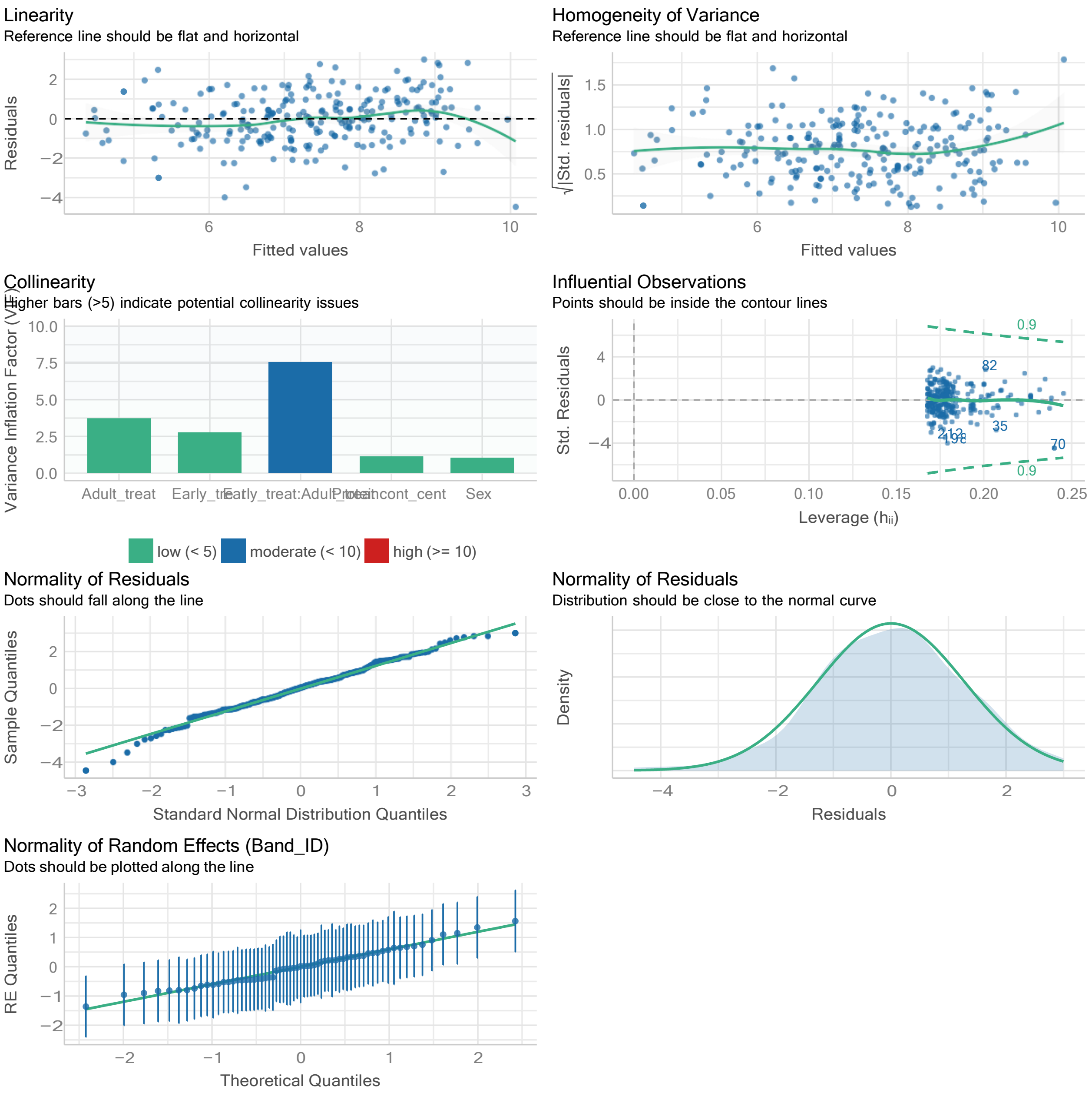

Figure 11: Model assumptions checks

Overall, the model behaves well and the fit to the data is good.

6.2.2 Effects

To evaluate predictors' statistical significance, we consider Wald F tests. Results are reported in Table 24.

Table 24: Analysis of Deviance Table (Type II Wald F tests with Kenward-Roger df)

Effects	F	Df	Df.res	Pr(>F)	
Proteincont_cent	39.14	1	214.17	2e-09	***
Sex	14.28	1	60.82	4e-04	***
Early_treat	6.87	3	58.15	5e-04	***
Adult_treat	0.10	1	180.89	0.749	
Early_treat:Adult_treat	4.03	3	173.68	0.008	**

Note: 0 '***' 0.001 '**' 0.01 '*' 0.05 '.' 0.1 ' ' 1

The interaction `Early_treat * Adult_treat` and the other effects are statistically significant.

We evaluate the effect by presenting the predicted values and post-hoc tests.

- **Protein Content.** Estimated effect is reported in Table 25 and presented in Figure 12.

Table 25: Estimated effects

Term	Estimate	Std. Error	2.5 %	97.5 %	df	t value	Pr(>|t|)	
Proteincont_cent	0.71	0.11	0.49	0.93	214.9	6.32	1e-09	***

Note: 0 '***' 0.001 '**' 0.01 '*' 0.05 '.' 0.1 ' ' 1

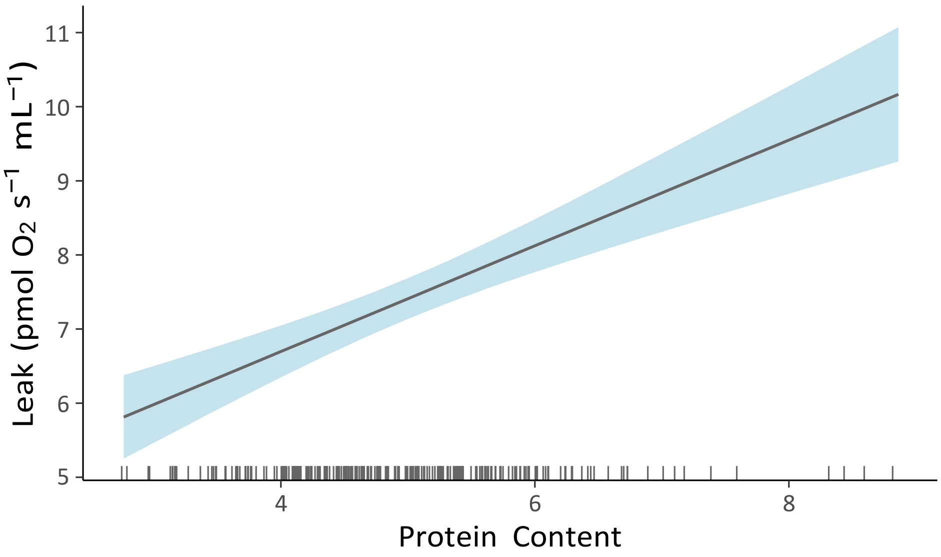

Figure 12: Protein Content effect

- **Sex.** Predicted values are reported in Table 26 and presented in Figure 13. Post hoc tests are reported in Table 27.

```
emmeans_sex <- emmeans::emmeans(model_leak, pairwise ~ Sex, adjust = "mvt")
```

Table 26: Predicted values

Sex	Predicted	SE	df	2.5%	97.5%
Female	7.97	0.22	60.42	7.54	8.40
Male	6.88	0.19	58.13	6.50	7.26

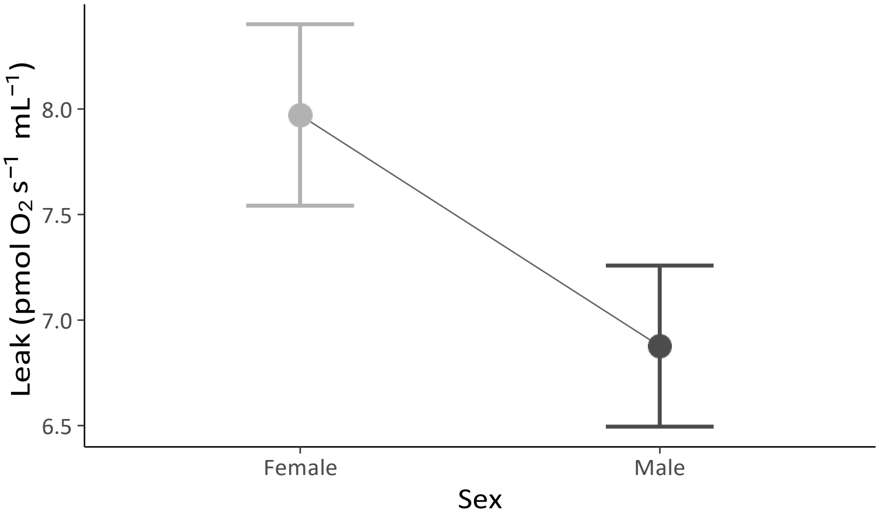

Figure 13: Sex predicted values

Table 27: Post hoc contrasts

Contrast	Estimate	SE	df	t.ratio	p.value	
Female - Male	1.09	0.29	60.82	3.78	4e-04	***

Note: 0 '***' 0.001 '**' 0.01 '*' 0.05 '.' 0.1 ' ' 1

- **Adult and Early Treatment Interaction.** Predicted values are reported in Table 28 and presented in Figure 14. Post hoc tests are reported in Table 29.

```
emmeans_int <- emmeans::emmeans(model_leak, ~ Early_treat * Adult_treat)
```

Table 28: Predicted values

Early Life Condition	Treatment in Adulthood	Predicted	SE	df	2.5%	97.5%
Constant 18C	Control 25C	6.82	0.31	117.49	6.21	7.43
Periodic 21C	Control 25C	8.48	0.31	106.48	7.87	9.09
Constant 35C	Control 25C	7.33	0.38	102.64	6.58	8.07
Periodic 40C	Control 25C	7.23	0.35	103.67	6.54	7.92
Constant 18C	Treat 40C	7.14	0.32	123.36	6.51	7.77
Periodic 21C	Treat 40C	8.27	0.33	124.20	7.61	8.93
Constant 35C	Treat 40C	6.18	0.38	106.76	5.43	6.93
Periodic 40C	Treat 40C	7.95	0.36	113.38	7.23	8.67

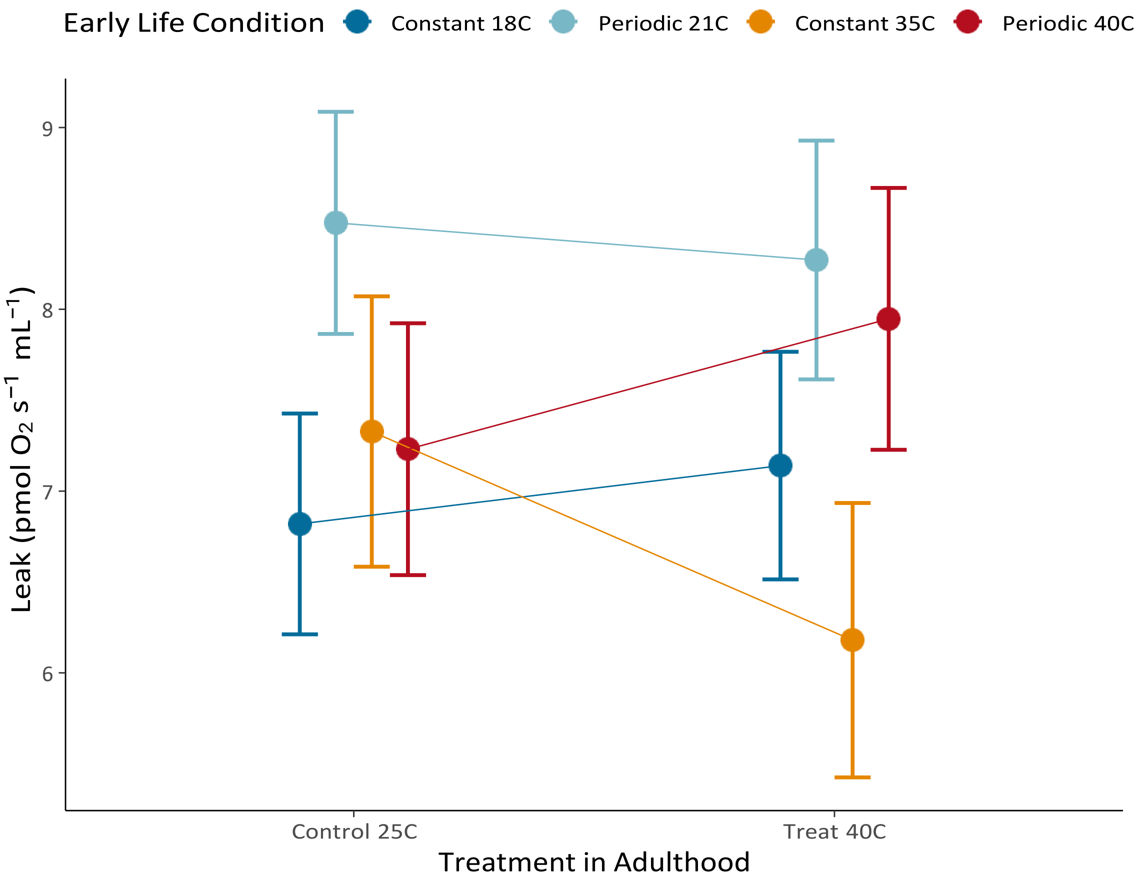

Figure 14: Interaction predicted values

Table 29: Post hoc contrasts

Contrast	Estimate	SE	df	t.ratio	p.value[1]	
**Contrasts in the Adult Control 25C**						
Early 18C - 21C	-1.66	0.43	112.55	-3.83	0.004	**
Early 18C - 35C	-0.51	0.48	108.03	-1.05	0.956	
Early 18C - 40C	-0.41	0.46	108.56	-0.89	0.982	
Early 21C - 35C	1.15	0.49	103.57	2.35	0.240	
Early 21C - 40C	1.25	0.46	105.50	2.69	0.116	
Early 35C - 40C	0.10	0.51	102.83	0.19	1.000	
**Contrasts in the Adult Treat 40C**						
Early 18C - 21C	-1.13	0.45	123.90	-2.49	0.179	
Early 18C - 35C	0.96	0.50	112.92	1.94	0.482	
Early 18C - 40C	-0.81	0.48	116.19	-1.69	0.653	
Early 21C - 35C	2.09	0.51	113.15	4.12	0.001	***
Early 21C - 40C	0.32	0.49	118.69	0.66	0.997	
Early 35C - 40C	-1.77	0.53	109.52	-3.35	0.019	*
**Within Group Differences[2]**						
Early 18C	0.32	0.36	182.95	0.89	0.982	
Early 21C	-0.20	0.35	179.72	-0.58	0.999	
Early 35C	-1.15	0.41	166.65	-2.80	0.086	.
Early 40C	0.72	0.39	173.14	1.82	0.561	
**Differences Between Within Group Differences[3]**						
Early 18C - 21C	0.53	0.50	177.22	1.05	0.956	
Early 18C - 35C	1.47	0.54	173.76	2.70	0.110	
Early 18C - 40C	-0.40	0.52	174.01	-0.76	0.993	
Early 21C - 35C	0.94	0.54	172.24	1.74	0.618	
Early 21C - 40C	-0.92	0.53	174.28	-1.75	0.613	
Early 35C - 40C	-1.86	0.57	169.76	-3.28	0.022	*

Note:

0 '***' 0.001 '**' 0.01 '*' 0.05 '.' 0.1 ' ' 1

[1] P value adjustment: mvt method for 22 tests

[2] Differences (Adult Treat 40C - Adult Control 25C) for each Early Life Condition

[3] Differences between slopes of each Early Life Condition

7 OxPhos Analysis

7.1 Model comparison

Results of the model comparison are reported below,

Table 30: Model comparison using AIC and BIC.

Model	Term	Df	AIC	$AIC_{weights}$	
m_0		5	1242.08	5%	
m_1	Early_treat	8	1245.66	1%	
m_2	Adult_treat	6	1236.61	78%	
m_3	Early_treat + Adult_treat	9	1240.25	13%	
m_4	Early_treat * Adult_treat	12	1242.89	3%	

Considering the AIC weights, model `m_2` (the model with only `Adult_treat`) is the best model (78%).

In the following analysis, we consider model `m_2`.

7.2 Selected Model

We re-estimate the selected model using the REML approach.

```
model_oxphos <- lmer(OxPhos ~ Adult_treat + Sex + Proteincont_cent + (1|Band_ID),
                     data = data_mito, REML = TRUE))
```

7.2.1 Fit and Checks

The summary of the model is presented in Table 31.

Table 31: Model summary

Term	Estimate	Std. Error	2.5 %	97.5 %	df	t value	Pr(>|t|)	
**Random Effets (sd)**								
Band_ID	1.88		1.26	2.45				
Residual	2.91		2.61	3.24				
**Fixed Effets**								
(Intercept)	20.14	0.49	19.17	21.10	77.97	40.70	<2e-16	***
Proteincont_cent	1.47	0.23	1.01	1.93	226.20	6.34	1e-09	***
SexMale	-0.82	0.62	-2.04	0.38	60.28	-1.33	0.188	
Adult_treatTreat 40C	-1.09	0.40	-1.89	-0.31	180.65	-2.73	0.007	**

Note: 0 '***' 0.001 '**' 0.01 '*' 0.05 '.' 0.1 ' ' 1

The fit of the model is presented in Table 32.

Table 32: Model fit

Marginal R^2	Conditional R^2
19.9%	43.4%

Model assumptions checks are presented in Figure 15.

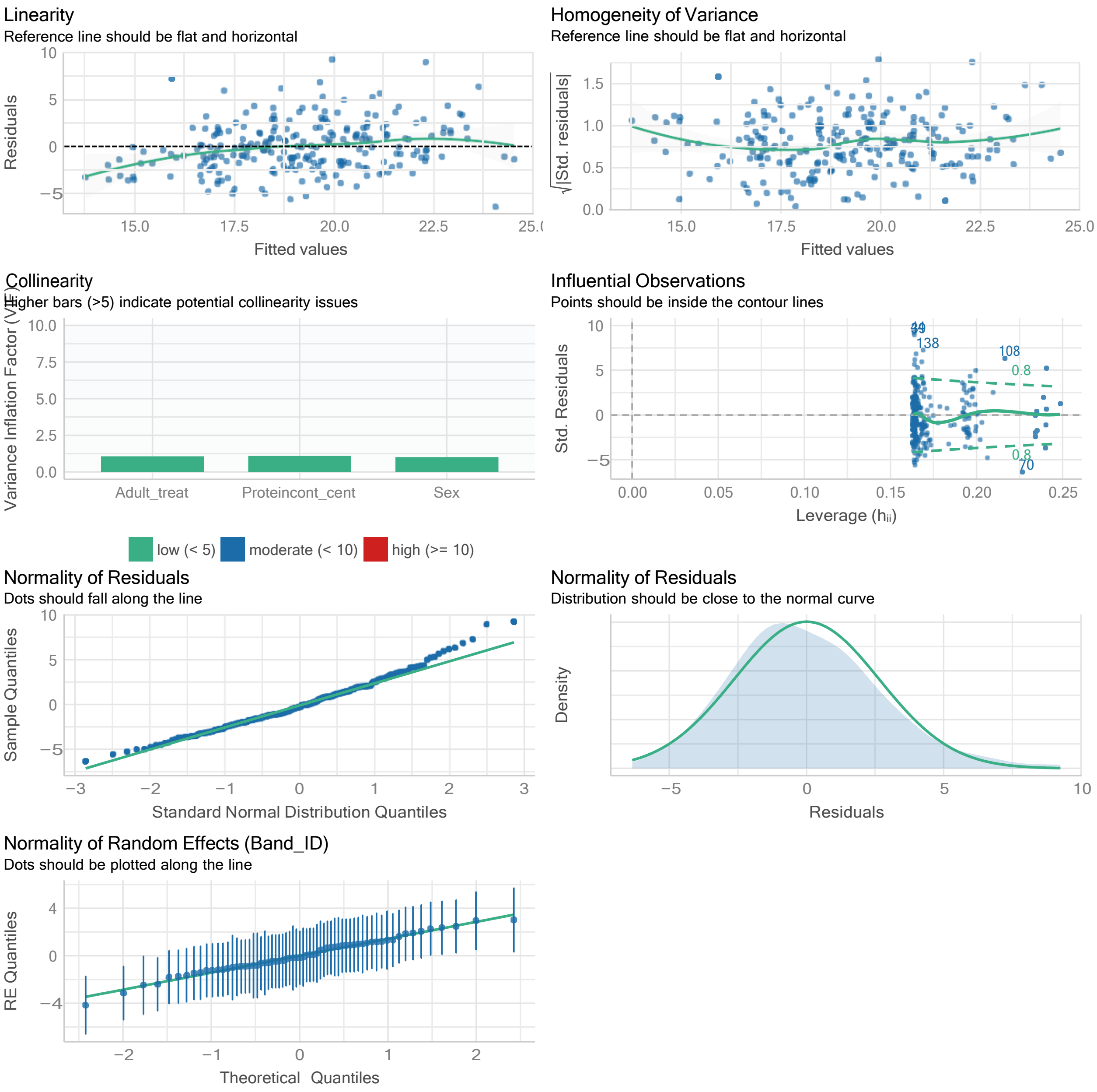

Figure 15: Model assumptions checks

Overall, the model behaves well and the fit to the data is good.

7.2.2 Effects

To evaluate predictors' statistical significance, we consider Wald F tests. Results are reported in Table 33.

Table 33: Analysis of Deviance Table (Type II Wald F tests with Kenward-Roger df)

Effects	F	Df	Df.res	Pr(>F)	
Proteincont_cent	39.40	1	226.54	2e-09	***
Sex	1.78	1	63.74	0.187	
Adult_treat	7.42	1	183.59	0.007	**

Note: 0 '***' 0.001 '**' 0.01 '*' 0.05 '.' 0.1 ' ' 1

Note that Sex is not statistically significant. On the contrary, Adult_treat and Proteincont_cent are statistically significant.

We evaluate the effect by presenting the predicted values and post-hoc tests.

- **Protein Content.** Estimated effect is reported in Table 34 and presented in Figure 16.

Table 34: Estimated effects

Term	Estimate	Std. Error	2.5 %	97.5 %	df	t value	Pr(>|t|)	
Proteincont_cent	1.47	0.23	1.01	1.93	226.2	6.34	1e-09	***

Note: 0 '***' 0.001 '**' 0.01 '*' 0.05 '.' 0.1 ' ' 1

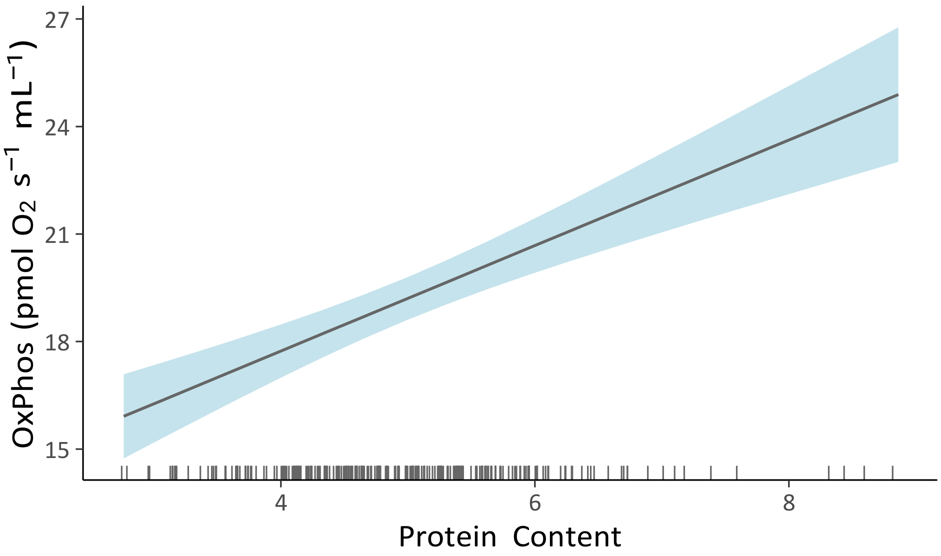

Figure 16: Protein Content effect

- **Sex.** Predicted values are reported in Table 35 and presented in Figure 17. Post hoc tests are reported

in Table 36.

```
emmeans_sex <- emmeans::emmeans(model_oxphos, pairwise ~ Sex, adjust = "mvt")
```

Table 35: Predicted values

Sex	Predicted	SE	df	2.5%	97.5%
Female	19.59	0.46	63.68	18.67	20.52
Male	18.77	0.40	62.08	17.97	19.57

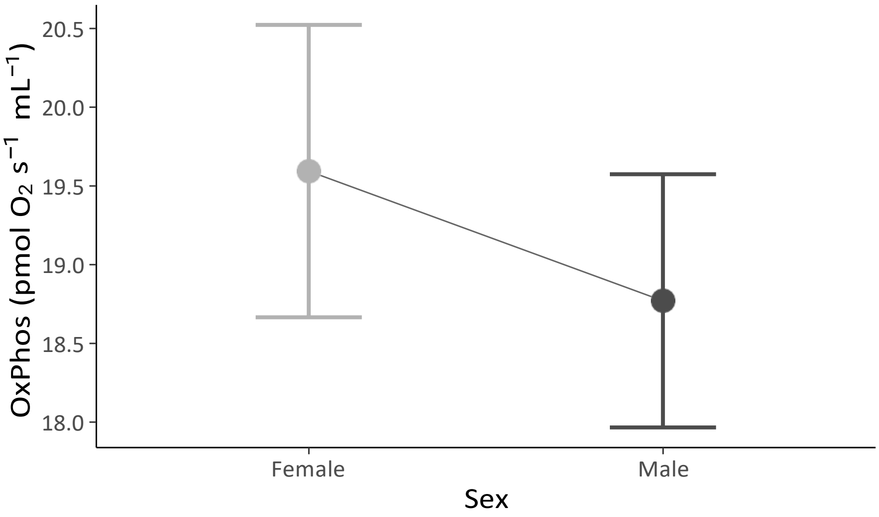

Figure 17: Sex predicted values

Table 36: Post hoc contrasts

Contrast	Estimate	SE	df	t.ratio	p.value
Female - Male	0.82	0.62	63.74	1.33	0.2

Note: 0 '***' 0.001 '**' 0.01 '*' 0.05 '.' 0.1 ' ' 1

- **Adult Treatment.** Predicted values are reported in Table 37 and presented in Figure 18. Post hoc tests are reported in Table 38.

```
emmeans_treat <- emmeans::emmeans(model_oxphos, pairwise ~ Adult_treat,
                                  adjust = "mvt")
```

Table 37: Predicted values

Treatment in Adulthood	Predicted	SE	df	2.5%	97.5%
Control 25C	19.73	0.36	109.87	19.02	20.44
Treat 40C	18.64	0.37	120.56	17.90	19.38

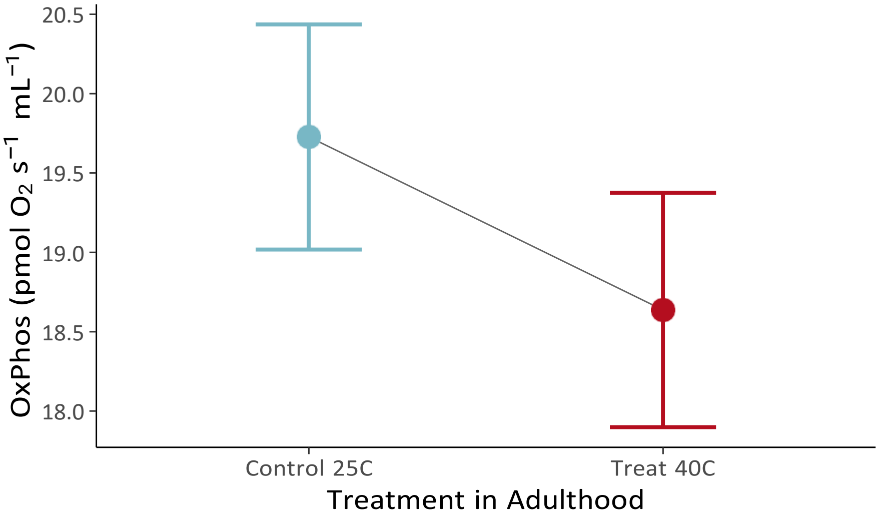

Figure 18: Adult Treatment predicted values

Table 38: Post hoc contrasts

Contrast	Estimate	SE	df	t.ratio	p.value	
(Adult 25C) - (Adult 40C)	1.09	0.4	183.59	2.72	0.007	**

Note: 0 '***' 0.001 '**' 0.01 '*' 0.05 '.' 0.1 ' ' 1

8 ETS Analysis

8.1 Model comparison

Results of the model comparison are reported below,

Table 39: Model comparison using AIC and BIC.

Model	Term	Df	AIC	$AIC_{weights}$
m_0		5	1922.87	14%
m_1	Early_treat	8	1923.71	9%
m_2	Adult_treat	6	1920.87	39%
m_3	Early_treat + Adult_treat	9	1921.55	28%
m_4	Early_treat * Adult_treat	12	1923.54	10%

Considering the AIC weights, model `m_2` (the model with only `Adult_treat`) is the best model (39%) These results indicate that there is some evidence regarding the `Adult_treat` effect.

In the following analyses, we consider `m_2` as it includes only the `Adult_treat` effect.

8.2 Selected Model

We re-estimate the selected model using the REML approach.

```
model_ets <- lmer(ETS ~ Adult_treat + Proteincont_cent + (1|Band_ID),
                  data = data_mito, REML = TRUE)
```

8.2.1 Fit and Checks

The summary of the model is presented in Table 40.

Table 40: Model summary

Term	Estimate	Std. Error	2.5 %	97.5 %	df	t value	Pr(>|t|)	
**Random Effets (sd)**								
Band_ID	7.93		5.28	10.33				
Residual	12.53		11.25	13.94				
**Fixed Effets**								
(Intercept)	82.68	2.11	78.57	86.78	79.70	39.25	<2e-16	***
Proteincont_cent	2.84	1.00	0.86	4.78	225.47	2.85	0.005	**
SexMale	-5.88	2.63	-11.01	-0.76	61.35	-2.24	0.029	*
Adult_treatTreat 40C	-3.41	1.72	-6.83	-0.07	181.80	-1.99	0.048	*

Note: 0 '***' 0.001 '**' 0.01 '*' 0.05 '.' 0.1 ' ' 1

The fit of the model is presented in Table 41.

Table 41: Model fit

Marginal R^2	Conditional R^2
8.1%	34.4%

Model assumptions checks are presented in Figure 19.

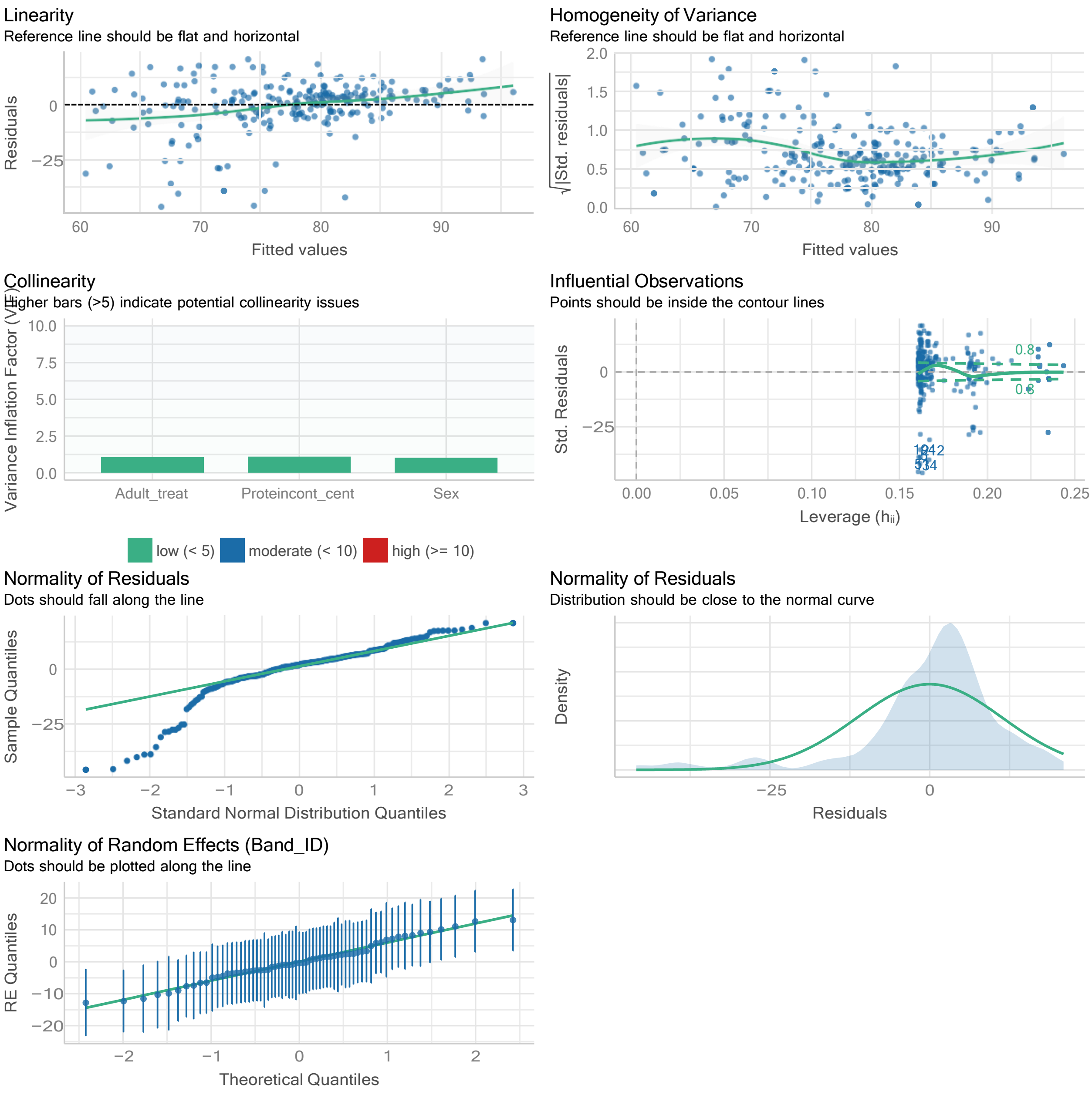

Figure 19: Model assumptions checks

The model is acceptable.

8.2.2 Effects

To evaluate predictors' statistical significance, we consider Wald F tests. Results are reported in Table 42.

Table 42: Analysis of Deviance Table (Type II Wald F tests with Kenward-Roger df)

Effects	F	Df	Df.res	Pr(>F)	
Proteincont_cent	7.97	1	225.74	0.005	**
Sex	5.01	1	63.73	0.029	*
Adult_treat	3.93	1	183.79	0.049	*

Note: 0 '***' 0.001 '**' 0.01 '*' 0.05 '.' 0.1 ' ' 1

The `Adult_treat` effect is not statistically significant but close to the statistical threshold.

We evaluate the effect by presenting the predicted values and post-hoc tests.

- **Protein Content.** Estimated effect is reported in Table 43 and presented in Figure 20.

Table 43: Estimated effects

Term	Estimate	Std. Error	2.5 %	97.5 %	df	t value	Pr(>|t|)	
Proteincont_cent	2.84	1	0.86	4.78	225.47	2.85	0.005	**

Note: 0 '***' 0.001 '**' 0.01 '*' 0.05 '.' 0.1 ' ' 1

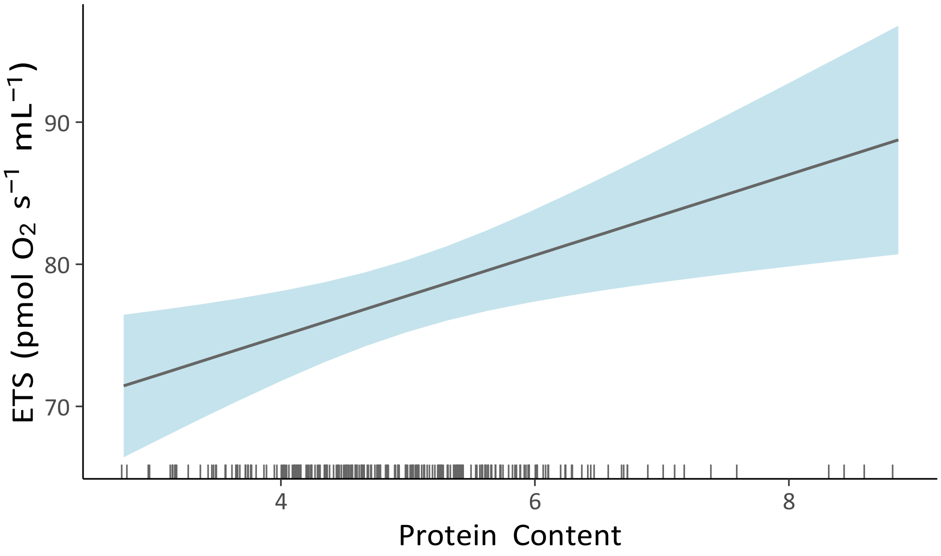

Figure 20: Protein Content effect

- **Sex.** Predicted values are reported in Table 44 and presented in Figure 21. Post hoc tests are reported in Table 45.

```r
emmeans_sex <- emmeans::emmeans(model_ets, pairwise ~ Sex, adjust = "mvt")
```

Table 44: Predicted values

Sex	Predicted	SE	df	2.5%	97.5%
Female	80.97	1.98	63.68	77.03	84.92
Male	75.09	1.71	62.03	71.68	78.51

Table 45: Post hoc contrasts

Contrast	Estimate	SE	df	t.ratio	p.value	
Female - Male	5.88	2.63	63.73	2.24	0.03	*

Note: 0 '***' 0.001 '**' 0.01 '*' 0.05 '.' 0.1 ' ' 1

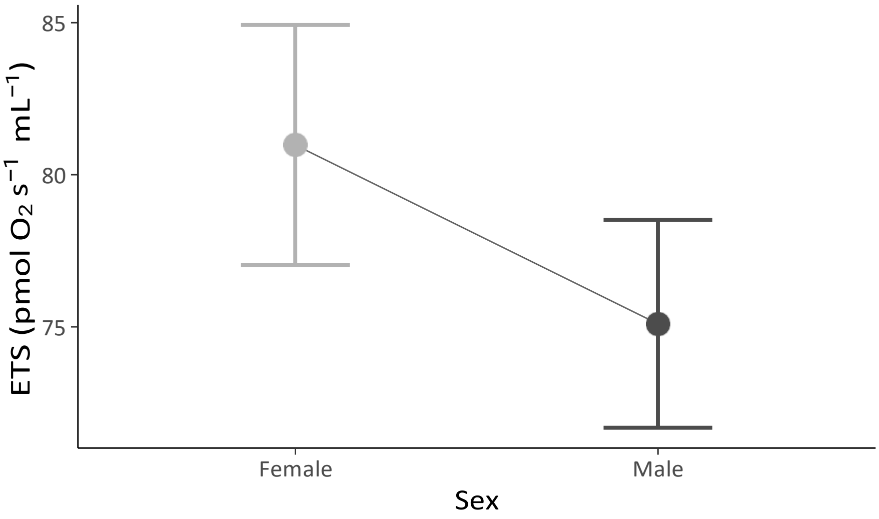

Figure 21: Sex predicted values

- **Adult Treatment.** Predicted values are reported in Table 46 and presented in Figure 22. Post hoc tests are reported in Table 47.

```
emmeans_treat <- emmeans::emmeans(model_ets, pairwise ~ Adult_treat, adjust = "mvt")
```

Table 46: Predicted values

Treatment in Adulthood	Predicted	SE	df	2.5%	97.5%
Control 25C	79.74	1.53	110.99	76.72	82.76
Treat 40C	76.33	1.59	121.74	73.18	79.48

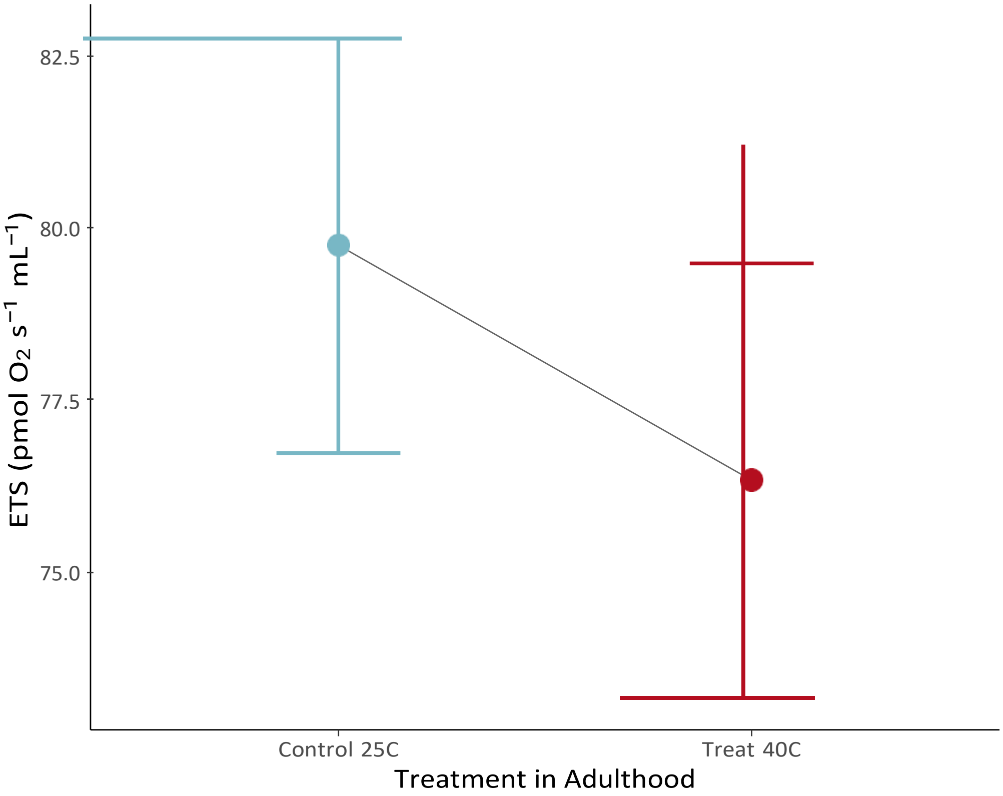

Figure 22: Interaction predicted values

Table 47: Post hoc contrasts

Contrast	Estimate	SE	df	t.ratio	p.value	
(Adult 25C) - (Adult 40C)	3.41	1.72	183.79	1.98	0.05	*

Note: 0 '***' 0.001 '**' 0.01 '*' 0.05 '.' 0.1 ' ' 1

9 OxCE Analysis

9.1 Model comparison

Note that for OxCE we removed `Proteincount` from all models.

Results of the model comparison are reported in Table 48.

Table 48: Model comparison using AIC and BIC.

Model	Term	Df	AIC	$AIC_{weights}$	
m_0		5	-744.79	4%	
m_1	Early_treat	8	-746.83	11%	
m_2	Adult_treat	6	-745.17	5%	
m_3	Early_treat + Adult_treat	9	-747.54	16%	
m_4	Early_treat * Adult_treat	12	-750.39	65%	

Considering the AIC weights, model `m_4` (the model with the interaction `Early_treat * Adult_treat`) is the best model (65%). Therefore, we can say that there is some evidence in favour of the interaction but we can not draw strong conclusions.

In the following analysis, we consider model `m_4`.

9.2 Selected Model

We re-estimate the selected model using the REML approach.

```
model_oxce <- lmer(OxCE ~ Early_treat*Adult_treat + Sex + (1|Band_ID),
                   data = data_mito, REML = TRUE)
```

9.2.1 Fit and Checks

The summary of the model is presented in Table 49.

Table 49: Model summary

Term	Estimate	Std. Error	2.5 %	97.5 %	df	t value	Pr(>|t|)	
**Random Effets (sd)**								
Band_ID	0.03		0.02	0.04				
Residual	0.04		0.04	0.05				
**Fixed Effets**								
(Intercept)	0.73	0.01	0.71	0.75	95.00	67.68	<2e-16	***
SexMale	0.02	0.01	0.00	0.04	59.61	2.33	0.023	*
Early_treatPeriodic 21C	-0.04	0.01	-0.06	-0.01	105.48	-2.87	0.005	**
Early_treatConstant 35C	-0.01	0.02	-0.04	0.01	101.52	-0.97	0.335	
Early_treatPeriodic 40C	-0.01	0.01	-0.04	0.02	102.40	-0.62	0.534	
Adult_treatTreat 40C	-0.02	0.01	-0.04	0.00	173.44	-1.92	0.057	.
Early_treatPeriodic 21C:Adult_treatTreat 40C	0.02	0.01	-0.01	0.05	175.69	1.41	0.162	
Early_treatConstant 35C:Adult_treatTreat 40C	0.03	0.02	0.00	0.07	169.70	2.17	0.031	*
Early_treatPeriodic 40C:Adult_treatTreat 40C	0.00	0.02	-0.03	0.03	172.51	-0.26	0.799	

Note: 0 '***' 0.001 '**' 0.01 '*' 0.05 '.' 0.1 ' ' 1

The fit of the model is presented in Table 50.

Table 50: Model fit

Marginal R^2	Conditional R^2
10.3%	38.4%

Model assumptions checks are presented in Figure 23.

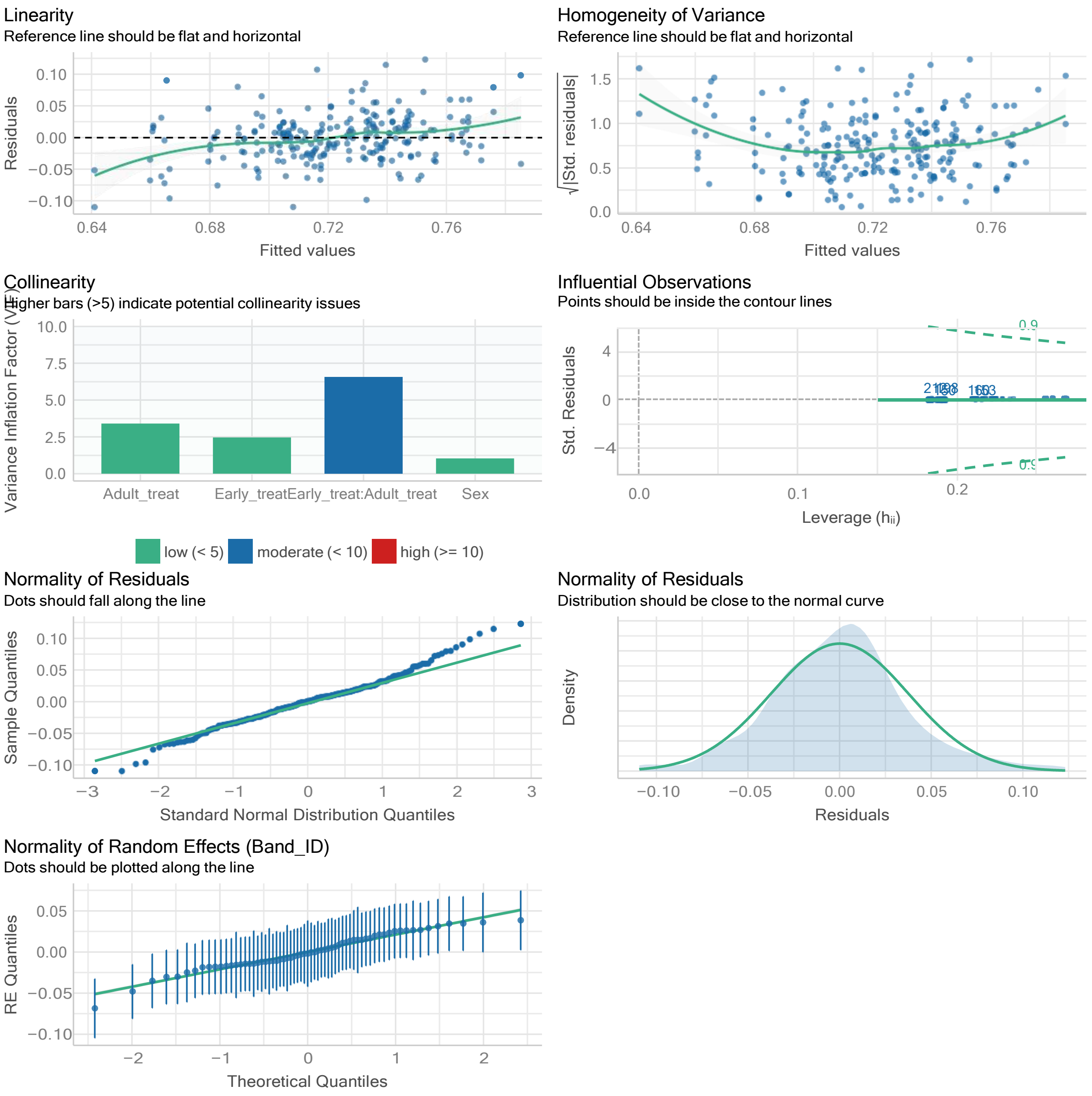

Figure 23: Model assumptions checks

Overall, the model behaves well but the fit to the data is not high.

9.2.2 Effects

To evaluate predictors' statistical significance, we consider Wald F tests. Results are reported in Table 51.

Table 51: Analysis of Deviance Table (Type II Wald F tests with Kenward-Roger df)

Effects	F	Df	Df.res	Pr(>F)	
Sex	5.43	1	59.84	0.02	*
Early_treat	2.69	3	58.84	0.05	.
Adult_treat	1.93	1	173.14	0.17	
Early_treat:Adult_treat	2.45	3	172.57	0.07	.

Note: 0 '***' 0.001 '**' 0.01 '*' 0.05 '.' 0.1 ' ' 1

Note that the interaction `Adult_treat:Early_treat` is not statistically significant but close to the statistical threshold. This is in line with the model comparison results that indicated some evidence in favor of the interaction but no strong conclusions can be drawn.

We evaluate the effect by presenting the predicted values and post-hoc tests.

- **Sex.** Predicted values are reported in Table 52 and presented in Figure 24. Post hoc tests are reported in Table 53.

```
emmeans_sex <- emmeans::emmeans(model_oxce, pairwise ~ Sex, adjust = "mvt")
```

Table 52: Predicted values

Sex	Predicted	SE	df	2.5%	97.5%
Female	0.71	0.01	60.04	0.70	0.72
Male	0.73	0.01	58.34	0.72	0.74

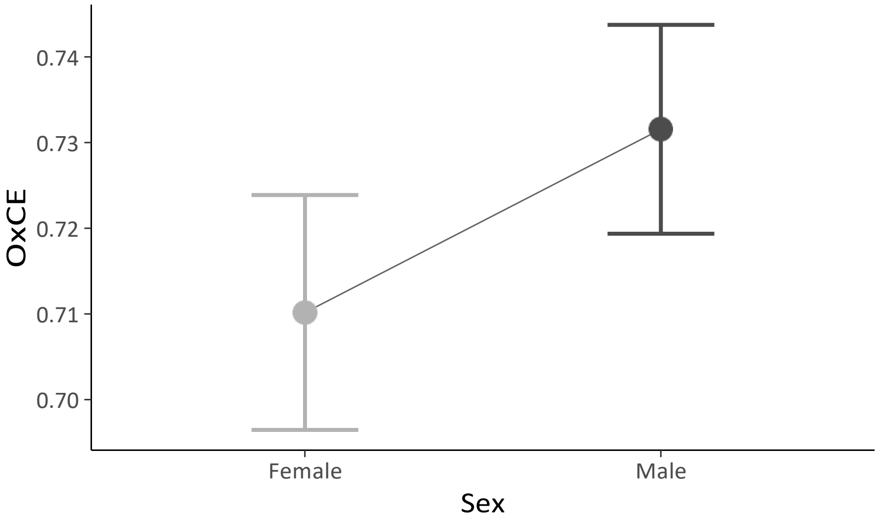

Figure 24: Sex predicted values

Table 53: Post hoc contrasts

Contrast	Estimate	SE	df	t.ratio	p.value	
Female - Male	-0.02	0.01	59.84	-2.33	0.02	*

Note: 0 '***' 0.001 '**' 0.01 '*' 0.05 '.' 0.1 ' ' 1

- **Adult and Early Treatment Interaction.** Predicted values are reported in Table 54 and presented in Figure 25. Post hoc tests are reported in Table 55.

```
emmeans_int <- emmeans::emmeans(model_oxce, ~ Early_treat * Adult_treat)
```

Table 54: Predicted values

Early Life Condition	Treatment in Adulthood	Predicted	SE	df	2.5%	97.5%
Constant 18C	Control 25C	0.74	0.01	109.78	0.72	0.76
Periodic 21C	Control 25C	0.70	0.01	100.94	0.68	0.72
Constant 35C	Control 25C	0.73	0.01	97.28	0.70	0.75
Periodic 40C	Control 25C	0.73	0.01	97.31	0.71	0.75
Constant 18C	Treat 40C	0.72	0.01	112.35	0.70	0.74
Periodic 21C	Treat 40C	0.70	0.01	118.29	0.68	0.72
Constant 35C	Treat 40C	0.74	0.01	101.06	0.72	0.76
Periodic 40C	Treat 40C	0.71	0.01	107.29	0.68	0.73

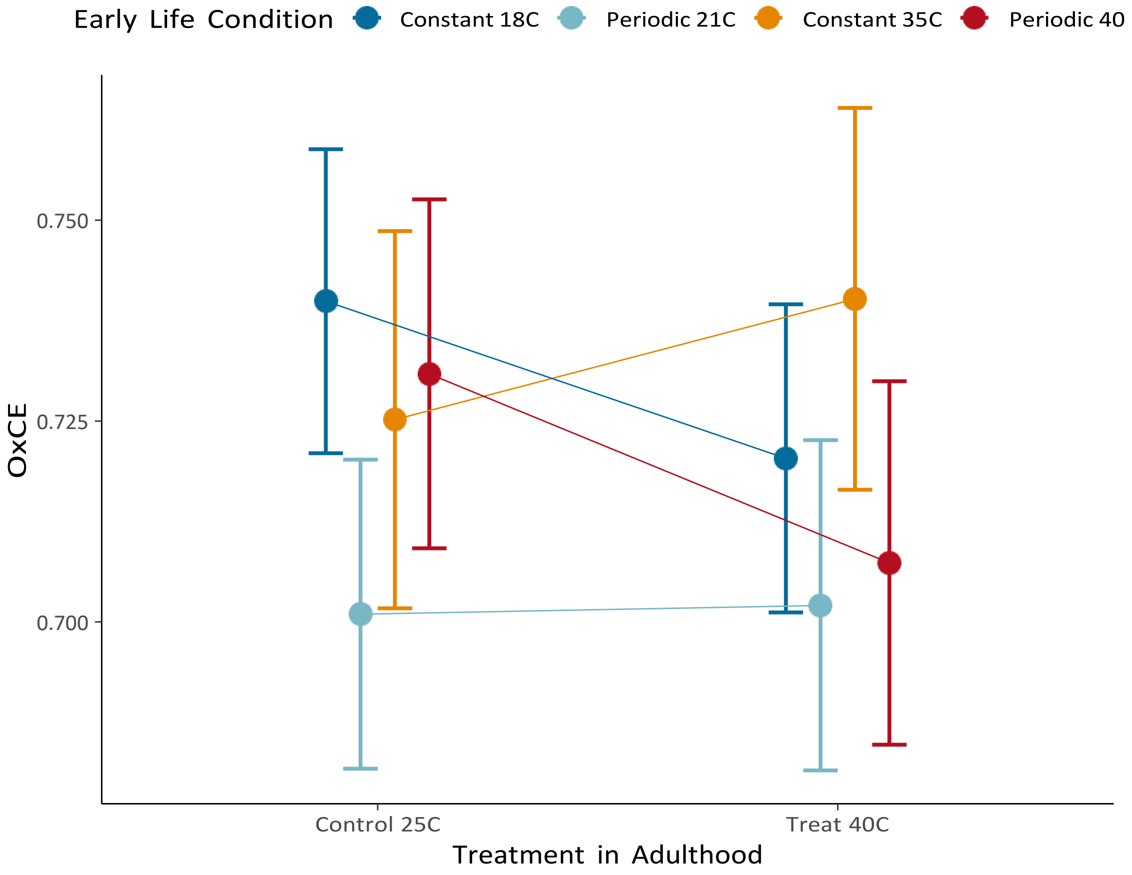

Figure 25: Interaction predicted values

Table 55: Post hoc contrasts

Contrast	Estimate	SE	df	t.ratio	p.value[1]	
**Contrasts in the Adult Control 25C**						
Early 18C - 21C	0.04	0.01	105.78	2.87	0.07	.
Early 18C - 35C	0.01	0.02	101.81	0.97	0.97	
Early 18C - 40C	0.01	0.01	102.69	0.62	1.00	
Early 21C - 35C	-0.02	0.02	98.21	-1.57	0.73	
Early 21C - 40C	-0.03	0.01	99.40	-2.06	0.41	
Early 35C - 40C	-0.01	0.02	97.11	-0.35	1.00	
**Contrasts in the Adult Treat 40C**						
Early 18C - 21C	0.02	0.01	115.83	1.29	0.88	
Early 18C - 35C	-0.02	0.02	105.17	-1.29	0.88	
Early 18C - 40C	0.01	0.01	109.56	0.87	0.98	
Early 21C - 35C	-0.04	0.02	107.40	-2.39	0.22	
Early 21C - 40C	-0.01	0.02	112.79	-0.34	1.00	
Early 35C - 40C	0.03	0.02	103.68	1.98	0.45	
**Within Group Differences[2]**						
Early 18C	-0.02	0.01	173.66	-1.91	0.50	
Early 21C	0.00	0.01	177.94	0.10	1.00	
Early 35C	0.02	0.01	167.29	1.23	0.90	
Early 40C	-0.02	0.01	172.02	-2.02	0.43	
**Differences Between Within Group Differences[3]**						
Early 18C - 21C	-0.02	0.01	175.90	-1.40	0.83	
Early 18C - 35C	-0.03	0.02	169.92	-2.17	0.33	
Early 18C - 40C	0.00	0.02	172.72	0.26	1.00	
Early 21C - 35C	-0.01	0.02	171.88	-0.86	0.99	
Early 21C - 40C	0.02	0.02	174.69	1.56	0.73	
Early 35C - 40C	0.04	0.02	169.53	2.28	0.27	

Note:

0 '***' 0.001 '**' 0.01 '*' 0.05 '.' 0.1 ' ' 1

[1] P value adjustment: mvt method for 22 tests

[2] Differences (Adult Treat 40C - Adult Control 25C) for each Early Life Condition

[3] Differences between slopes of each Early Life Condition

10 FCR$_5$ Analysis

10.1 Model comparison

Note that for FCR$_5$ we removed `Proteincount` from all models.

Results of the model comparison are reported in Table 56.

Table 56: Model comparison using AIC and BIC.

Model	Term	Df	AIC	$AIC_{weights}$
m_0		5	-328.73	9%
m_1	Early_treat	8	-328.69	9%
m_2	Adult_treat	6	-327.07	4%
m_3	Early_treat + Adult_treat	9	-327.11	4%
m_4	Early_treat * Adult_treat	12	-332.84	73%

Considering the AIC weights, model `m_4` (the model with the interaction `Early_treat * Adult_treat`) is the best model (77%). Therefore, we can say that there is some evidence in favor of the interaction but we can not draw strong conclusions.

In the following analysis, we consider model `m_4`.

10.2 Selected Model

We re-estimate the selected model using the REML approach.

```
model_fcr <- lmer(FCR ~ Early_treat * Adult_treat + Sex + (1|Band_ID),
                  data = data_mito, REML = TRUE)
```

10.2.1 Fit and Checks

Summary of the model is presented in Table 57.

Table 57: Model summary

Term	Estimate	Std. Error	2.5 %	97.5 %	df	t value	Pr(>|t|)	
**Random Effets (sd)**								
Band_ID	0.04		0.00	0.06				
Residual	0.11		0.10	0.12				
**Fixed Effets**								
(Intercept)	0.38	0.02	0.34	0.43	125.25	16.93	<2e-16	***
SexMale	0.01	0.02	-0.02	0.04	60.25	0.58	0.562	
Early_treatPeriodic 21C	-0.03	0.03	-0.09	0.02	143.72	-1.09	0.278	
Early_treatConstant 35C	-0.05	0.03	-0.11	0.01	138.89	-1.52	0.131	
Early_treatPeriodic 40C	-0.07	0.03	-0.13	-0.01	140.16	-2.23	0.027	*
Adult_treatTreat 40C	-0.06	0.03	-0.11	-0.01	178.29	-2.17	0.032	*
Early_treatPeriodic 21C:Adult_treatTreat 40C	0.11	0.04	0.04	0.19	181.35	2.88	0.004	**
Early_treatConstant 35C:Adult_treatTreat 40C	0.02	0.04	-0.06	0.11	173.13	0.57	0.567	
Early_treatPeriodic 40C:Adult_treatTreat 40C	0.11	0.04	0.03	0.19	176.76	2.79	0.006	**

Note: 0 '***' 0.001 '**' 0.01 '*' 0.05 '.' 0.1 ' ' 1

Fit of the model is presented in Table 58.

Table 58: Model fit

Marginal R^2	Conditional R^2
7.9%	17.2%

Model assumptions checks are presented in Figure 26.

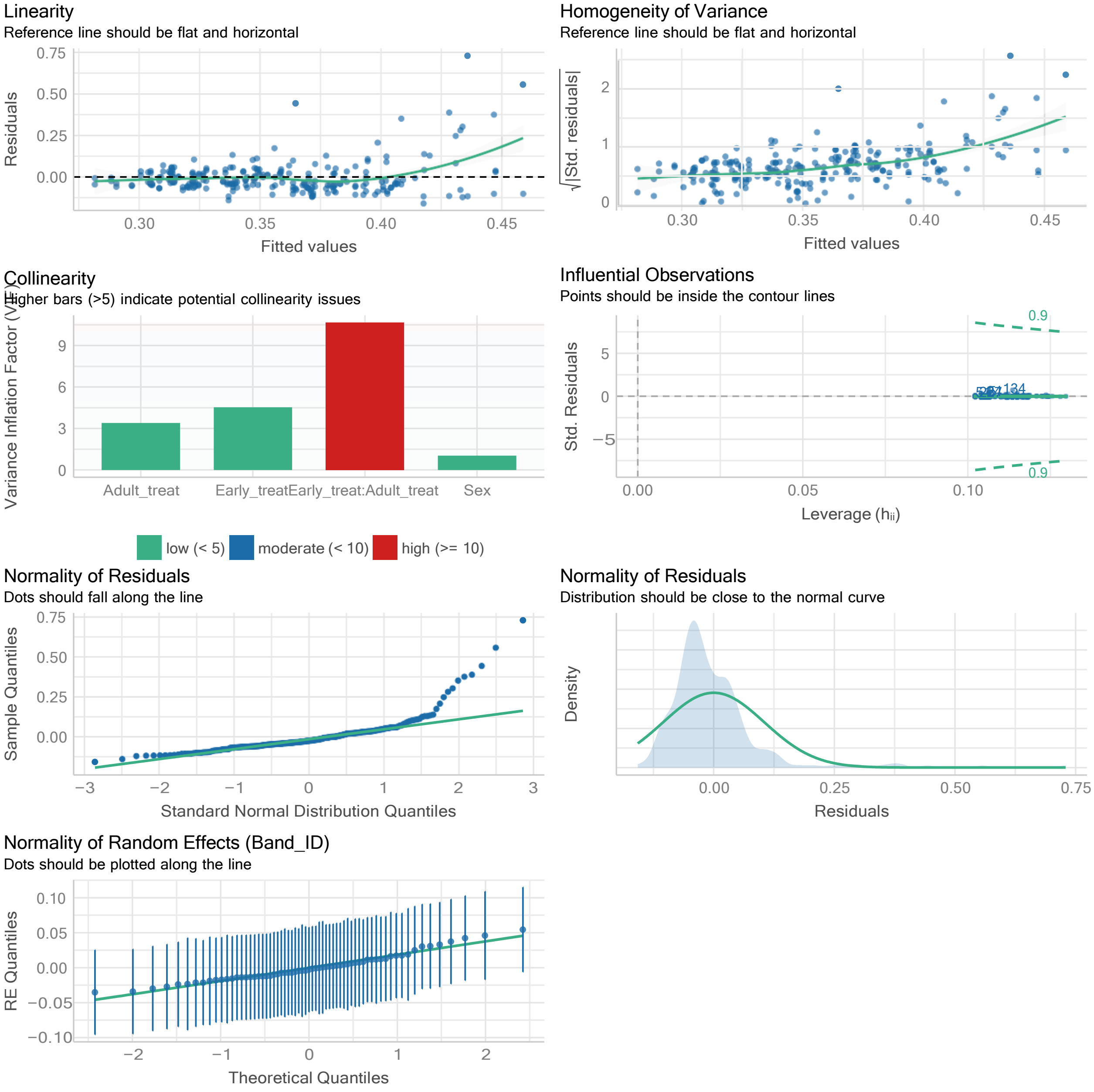

Figure 26: Model assumptions checks

The model is acceptable.

10.2.2 Effects

To evaluate predictors' statistical significance, we consider Wald F tests. Results are reported in Table 59.

Table 59: Analysis of Deviance Table (Type II Wald F tests with Kenward-Roger df)

Effects	F	Df	Df.res	Pr(>F)	
Sex	0.34	1	59.30	0.562	
Early_treat	1.84	3	57.76	0.151	
Adult_treat	0.05	1	176.66	0.828	
Early_treat:Adult_treat	4.22	3	175.82	0.007	**

Note: 0 '***' 0.001 '**' 0.01 '*' 0.05 '.' 0.1 ' ' 1

The interaction `Adult_treat:Early_treat` is statistically significant but `Sex` is not.

We evaluate the effect by presenting the predicted values and post-hoc tests.

- **Sex.** Predicted values are reported in Table 60 and presented in Figure 27. Post hoc tests are reported in Table 61.

```
emmeans_sex <- emmeans::emmeans(model_fcr, pairwise ~ Sex, adjust = "mvt")
```

Table 60: Predicted values

Sex	Predicted	SE	df	2.5%	97.5%
Female	0.35	0.01	59.89	0.32	0.38
Male	0.36	0.01	56.73	0.34	0.38

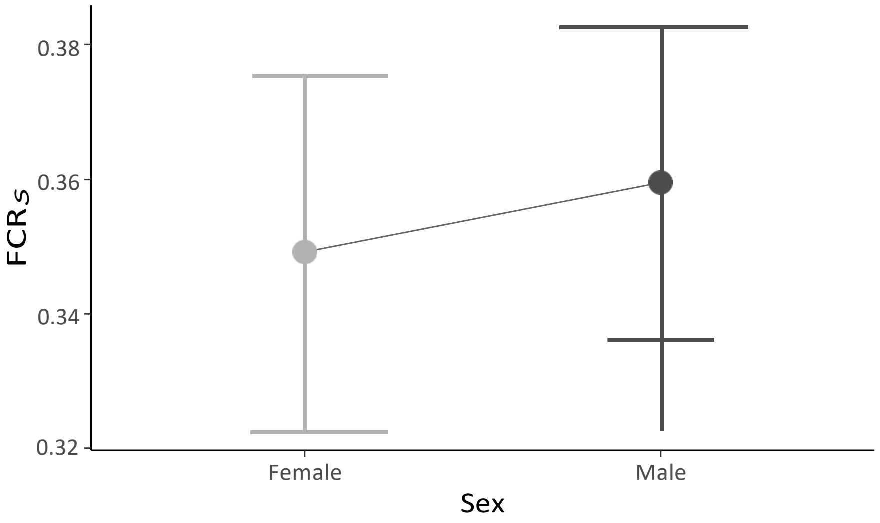

Figure 27: Sex predicted values

Table 61: Post hoc contrasts

Contrast	Estimate	SE	df	t.ratio	p.value
Female - Male	-0.01	0.02	59.3	-0.58	0.6

Note: 0 '***' 0.001 '**' 0.01 '*' 0.05 '.' 0.1 ' ' 1

- **Adult and Early Treatment Interaction.** Predicted values are reported in Table 62 and presented in Figure 28. Post hoc tests are reported in Table 63.

```
emmeans_int <- emmeans::emmeans(model_fcr, ~ Early_treat * Adult_treat)
```

Table 62: Predicted values

Early Life Condition	Treatment in Adulthood	Predicted	SE	df	2.5%	97.5%
Constant 18C	Control 25C	0.39	0.02	147.50	0.35	0.43
Periodic 21C	Control 25C	0.36	0.02	135.98	0.32	0.40
Constant 35C	Control 25C	0.34	0.03	131.95	0.29	0.39
Periodic 40C	Control 25C	0.32	0.02	132.01	0.27	0.37
Constant 18C	Treat 40C	0.33	0.02	147.77	0.29	0.37
Periodic 21C	Treat 40C	0.41	0.02	151.20	0.37	0.46
Constant 35C	Treat 40C	0.31	0.03	136.71	0.26	0.36
Periodic 40C	Treat 40C	0.38	0.02	140.57	0.33	0.42

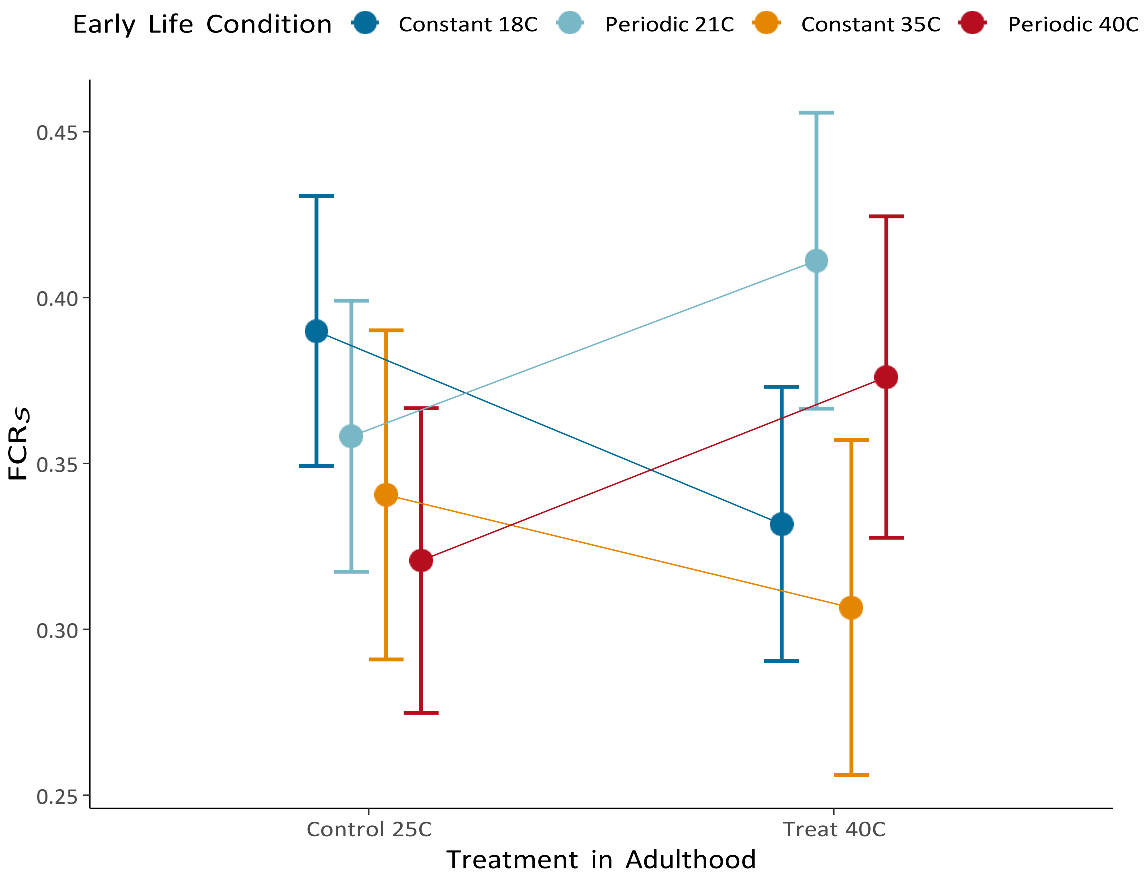

Figure 28: Interaction predicted values

Table 63: Post hoc contrasts

Contrast	Estimate	SE	df	t.ratio	p.value[1]	
**Contrasts in the Adult Control 25C**						
Early 18C - 21C	0.03	0.03	142.58	1.09	0.94	
Early 18C - 35C	0.05	0.03	137.73	1.52	0.75	
Early 18C - 40C	0.07	0.03	139.00	2.23	0.29	
Early 21C - 35C	0.02	0.03	132.63	0.54	1.00	
Early 21C - 40C	0.04	0.03	134.65	1.21	0.91	
Early 35C - 40C	0.02	0.03	131.63	0.58	1.00	
**Contrasts in the Adult Treat 40C**						
Early 18C - 21C	-0.08	0.03	149.95	-2.59	0.14	
Early 18C - 35C	0.03	0.03	140.80	0.76	0.99	
Early 18C - 40C	-0.04	0.03	143.77	-1.38	0.83	
Early 21C - 35C	0.10	0.03	141.80	3.06	0.04	*
Early 21C - 40C	0.04	0.03	146.18	1.06	0.95	
Early 35C - 40C	-0.07	0.04	138.15	-1.96	0.46	
**Within Group Differences[2]**						
Early 18C	-0.06	0.03	177.47	-2.16	0.33	
Early 21C	0.05	0.03	183.36	1.91	0.49	
Early 35C	-0.03	0.03	168.62	-1.05	0.95	
Early 40C	0.06	0.03	174.75	1.81	0.56	
**Differences Between Within Group Differences[3]**						
Early 18C - 21C	-0.11	0.04	180.57	-2.88	0.07	.
Early 18C - 35C	-0.02	0.04	172.25	-0.57	1.00	
Early 18C - 40C	-0.11	0.04	175.93	-2.78	0.09	.
Early 21C - 35C	0.09	0.04	174.91	2.04	0.40	
Early 21C - 40C	0.00	0.04	178.64	-0.06	1.00	
Early 35C - 40C	-0.09	0.04	171.51	-2.00	0.43	

Note:

0 '***' 0.001 '**' 0.01 '*' 0.05 '.' 0.1 ' ' 1

[1] P value adjustment: mvt method for 22 tests

[2] Differences (Adult Treat 40C - Adult Control 25C) for each Early Life Condition

[3] Differences between slopes of each Early Life Condition

11 Extra

Evaluating Adult Treatment effect on Routine, ETS, and OxPhos outcomes in Figure 29.

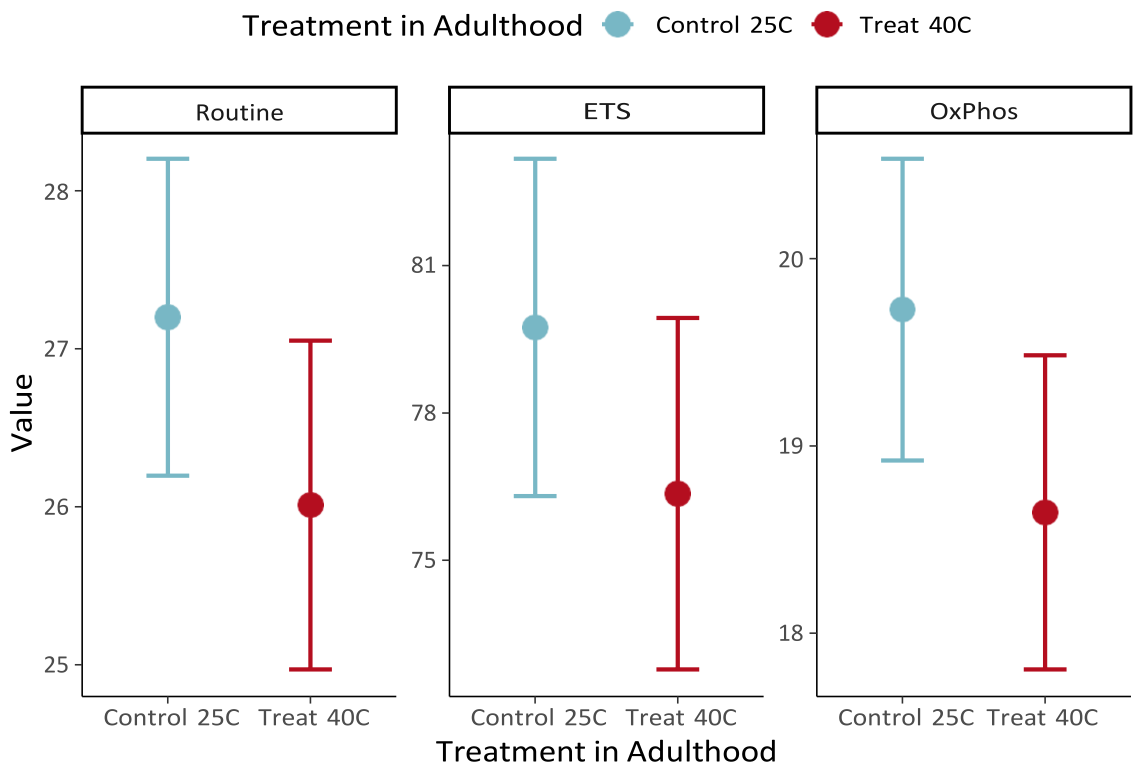

Figure 29: Adult Treatment effect on Routine, ETS, and OxPhos outcomes

References

Akaike, H. (1973). Information theory and an extension of the maximum likelihood principle. In *Second International Symposium on Information Theory* (pp. 267–281). Petrov, B. N. and Csaki, F., editors.

Bartón, K. (2019). MuMIn: Multi-model inference. R package version 1.43.6 Available at: https://cran.r-project.org/web/packages/MuMIn/ [Accessed January 13, 2022].

Fox, J. (2015). *Applied regression analysis and generalized linear models*. 3rd ed. Sage Publications.

Lenth, R. V. (2021). emmeans: Estimated Marginal Means, aka Least-Squares Means. R package version 1.7.1-1. Available at: https://CRAN.R-project.org/package=emmeans [Accessed November 20, 2021].

Lüdecke, D., Ben-Shachar, M., Patil, I., Waggoner, P., and Makowski, D. (2021). performance: An R Package for Assessment, Comparison and Testing of Statistical Models. J. Open Source Softw. 6, 3139. doi:10.21105/joss.03139.

McElreath, R. (2020). *Statistical rethinking: A Bayesian course with examples in R and Stan.* 2nd ed. Taylor and Francis.

R Core Team. (2021). *R: A language and environment for statistical computing* [Manual]. R Foundation for Statistical Computing. https://www.R-project.org/

Vandekerckhove, J., Matzke, D., & Wagenmakers, E.-J. (2015). Model Comparison and the Principle of Parsimony. In J. R. Busemeyer, Z. Wang, J. T. Townsend, & A. Eidels (Eds.), *The Oxford Handbook of Computational and Mathematical Psychology* (Vol. 1). Oxford University Press. https://doi.org/10.1093/oxfordhb/9780199957996.013.14

Wagenmakers, E.-J., & Farrell, S. (2004). AIC model selection using Akaike weights. *Psychonomic Bulletin & Review*, *11*(1), 192–196. https://doi.org/10.3758/BF03206482
