# Supplementary material for: Short- and long-term consequences of heat exposure on mitochondrial metabolism in zebra finches (Taeniopygia castanotis)
Source: Oecologia. 2023 Mar 10;201(3):637–48. doi: 10.1007/s00442-023-05344-7 (PMC10038956; doi:10.1007/s00442-023-05344-7)
Supplement: Supplementary file 2 — Supplementary file2 (PDF 957 KB) [file 442_2023_5344_MOESM2_ESM.pdf]

Appendix A

Routine Analysis

Table 1: Model comparison using AIC and BIC for Routine outcome.

Model	Term	Df	AIC	$AIC_{weights}$	
m_0		5	1349.83	2%	
m_1	Early_treat	8	1347.18	6%	
m_2	Adult_treat	6	1346.42	9%	
m_3	Early_treat + Adult_treat	9	1343.77	32%	
m_4	Early_treat * Adult_treat	12	1342.85	51%	

Table 2: Analysis of Deviance Table (Type II Wald F tests with Kenward-Roger df) for Routine outcome.

Effects	F	Df	Df.res	Pr(>F)	
Proteincont_cent	54.15	1	216.56	4e-12	***
Sex	7.26	1	60.85	0.009	**
Early_treat	2.79	3	58.24	0.048	*
Adult_treat	5.30	1	180.47	0.022	*
Early_treat:Adult_treat	2.31	3	173.40	0.078	.

Note: 0 '***' 0.001 '**' 0.01 '*' 0.05 '.' 0.1 ' ' 1

Leak Analysis

Table 3: Model comparison using AIC and BIC for Leak outcome.

Model	Term	Df	AIC	$AIC_{weights}$	
m_0		5	910.47	0%	
m_1	Early_treat	8	896.99	11%	
m_2	Adult_treat	6	912.32	0%	
m_3	Early_treat + Adult_treat	9	898.88	4%	
m_4	Early_treat * Adult_treat	12	892.86	85%	

Table 4: Analysis of Deviance Table (Type II Wald F tests with Kenward-Roger df) for Leak outcome.

Effects	F	Df	Df.res	Pr(>F)	
Proteincont_cent	39.14	1	214.17	2e-09	***
Sex	14.28	1	60.82	4e-04	***
Early_treat	6.87	3	58.15	5e-04	***
Adult_treat	0.10	1	180.89	0.749	
Early_treat:Adult_treat	4.03	3	173.68	0.008	**

Note: 0 ‘***’ 0.001 ‘**’ 0.01 ‘*’ 0.05 ‘.’ 0.1 ‘ ’ 1

OxPhos Analysis

Table 5: Model comparison using AIC and BIC for OxPhos outcome.

Model	Term	Df	AIC	$AIC_{weights}$	
m_0		5	1242.08	5%	
m_1	Early_treat	8	1245.66	1%	
m_2	Adult_treat	6	1236.61	78%	
m_3	Early_treat + Adult_treat	9	1240.25	13%	
m_4	Early_treat * Adult_treat	12	1242.89	3%	

Table 6: Analysis of Deviance Table (Type II Wald F tests with Kenward-Roger df) for OxPhos outcome.

Effects	F	Df	Df.res	Pr(>F)	
Proteincont_cent	39.40	1	226.54	2e-09	***
Sex	1.78	1	63.74	0.187	
Adult_treat	7.42	1	183.59	0.007	**

Note: 0 '***' 0.001 '**' 0.01 '*' 0.05 '.' 0.1 ' ' 1

ETS Analysis

Table 7: Model comparison using AIC and BIC for ETS outcome.

Model	Term	Df	AIC	AIC$_{weights}$
m_0		5	1922.87	14%
m_1	Early_treat	8	1923.71	9%
m_2	Adult_treat	6	1920.87	39%
m_3	Early_treat + Adult_treat	9	1921.55	28%
m_4	Early_treat * Adult_treat	12	1923.54	10%

Table 8: Analysis of Deviance Table (Type II Wald F tests with Kenward-Roger df) for ETS outcome.

Effects	F	Df	Df.res	Pr(>F)	
Proteincont_cent	7.97	1	225.74	0.005	**
Sex	5.01	1	63.73	0.029	*
Adult_treat	3.93	1	183.79	0.049	*

Note: 0 '***' 0.001 '**' 0.01 '*' 0.05 '.' 0.1 ' ' 1

OxCE Analysis

Table 9: Model comparison using AIC and BIC for OxCE outcome.

Model	Term	Df	AIC	$AIC_{weights}$	
m_0		5	-744.79	4%	
m_1	Early_treat	8	-746.83	11%	
m_2	Adult_treat	6	-745.17	5%	
m_3	Early_treat + Adult_treat	9	-747.54	16%	
m_4	Early_treat * Adult_treat	12	-750.39	65%	

Table 10: Analysis of Deviance Table (Type II Wald F tests with Kenward-Roger df) for OxCE outcome.

Effects	F	Df	Df.res	Pr(>F)	
Sex	5.43	1	59.84	0.02	*
Early_treat	2.69	3	58.84	0.05	.
Adult_treat	1.93	1	173.14	0.17	
Early_treat:Adult_treat	2.45	3	172.57	0.07	.

Note: 0 '***' 0.001 '**' 0.01 '*' 0.05 '.' 0.1 ' ' 1

FCR_S Analysis

Table 11: Model comparison using AIC and BIC for FCR_S outcome.

Model	Term	Df	AIC	$AIC_{weights}$	
m_0		5	-328.73	9%	
m_1	Early_treat	8	-328.69	9%	
m_2	Adult_treat	6	-327.07	4%	
m_3	Early_treat + Adult_treat	9	-327.11	4%	
m_4	Early_treat * Adult_treat	12	-332.84	73%	

Table 12: Analysis of Deviance Table (Type II Wald F tests with Kenward-Roger df) for FCR_S outcome.

Effects	F	Df	Df.res	Pr(>F)	
Sex	0.34	1	59.30	0.562	
Early_treat	1.84	3	57.76	0.151	
Adult_treat	0.05	1	176.66	0.828	
Early_treat:Adult_treat	4.22	3	175.82	0.007	**

Note: 0 '***' 0.001 '**' 0.01 '*' 0.05 '.' 0.1 ' ' 1
